# CDK12 and Integrator-PP2A complex modulates LEO1 phosphorylation for processive transcription elongation

**DOI:** 10.1126/sciadv.adf8698

**Published:** 2023-05-19

**Authors:** Min Qiu, Zhinang Yin, Honghong Wang, Lingyu Lei, Conghui Li, Yali Cui, Rong Dai, Peiyuan Yang, Ying Xiang, Qiuzi Li, Junhui Lv, Zhuang Hu, Min Chen, Hai-Bing Zhou, Pingping Fang, Ruijing Xiao, Kaiwei Liang

**Affiliations:** ^1^ Hubei Province Key Laboratory of Allergy and Immunology, School of Basic Medical Sciences, Wuhan University, Wuhan 430071, China.; ^2^ State Key Laboratory of Virology, Frontier Science Center for Immunology and Metabolism, Hubei Province Engineering and Technology Research Center for Fluorinated Pharmaceuticals, School of Pharmaceutical Sciences, Wuhan University, Wuhan 430071, China.; ^3^ Department of Rheumatology and Immunology, Zhongnan Hospital, Wuhan University, Wuhan 430071, China.; ^4^ TaiKang Center for Life and Medical Sciences, TaiKang Medical School, Wuhan University, Wuhan 430071, China.

## Abstract

Cyclin-dependent kinase 12 (CDK12) interacts with cyclin K to form a functional nuclear kinase that promotes processive transcription elongation through phosphorylation of the C-terminal domain of RNA polymerase II (Pol II). To gain a comprehensive understanding of CDK12's cellular function, we used chemical genetic and phosphoproteomic screening to identify a landscape of nuclear human CDK12 substrates, including regulators of transcription, chromatin organization, and RNA splicing. We further validated LEO1, a subunit of the polymerase-associated factor 1 complex (PAF1C), as a bona fide cellular substrate of CDK12. Acute depletion of LEO1, or substituting LEO1 phosphorylation sites with alanine, attenuated PAF1C association with elongating Pol II and impaired processive transcription elongation. Moreover, we discovered that LEO1 interacts with and is dephosphorylated by the Integrator-PP2A complex (INTAC) and that INTAC depletion promotes the association of PAF1C with Pol II. Together, this study reveals an uncharacterized role for CDK12 and INTAC in regulating LEO1 phosphorylation, providing important insights into gene transcription and its regulation.

## Teaser

CDK12 phosphorylates the LEO1 subunit of PAF1 complex to promote transcription elongation.

## INTRODUCTION

RNA polymerase II (Pol II)–mediated gene transcription in metazoans is a highly orchestrated and complex process, including transcription initiation as well as promoter-proximal pausing, elongation, and termination, and it is tightly regulated at these steps by a large number of proteins (*1*, *2*). The C-terminal domain (CTD) of the largest subunit of Pol II forms a flexible tail-like extension from the catalytic core of Pol II, serving as an important phosphorylation-regulated platform for gene transcriptional regulation (*3*, *4*). The CTD coordinates the transcription cycle through interaction with a wide range of factors and undergoes a cycle of phosphorylation and dephosphorylation by kinases and phosphatases during the transcription cycle (*5*, *6*). *Drosophila* cyclin-dependent kinase 12 (CDK12) (*7*) and human homologs CDK12 and CDK13 interact with cyclin K (CCNK) (*8*, *9*) to form functional nuclear complexes that act as transcriptional elongation-stage CTD kinases (*10*, *11*).

CDK12 is generally believed to catalyze the phosphorylation of CTD and promote Pol II–mediated transcription elongation in cooperation with CDK13 (*12*, *13*). Phenotypic outcomes linked with CDK12 depletion or enzymatic inhibition include decreased elongation rates and premature termination represented by selective loss of Pol II toward gene ends (*12*, *14*–*16*), which are usually attributed to changes in CTD phosphorylation. However, emerging evidence suggests that global CTD phosphorylation is mildly altered by CDK12 in some cellular contexts (*10*), implying that Pol II CTD may not be the sole substrate of CDK12. CDK12 can also associate with RNA processing factors (*11*, *17*, *18*) and regulate cotranscriptional RNA splicing and polyadenylation (*16*, *19*). Moreover, CDK12 can phosphorylate the mRNA 5′ cap-binding repressor 4E-BP1 to promote a specialized translation network including encoding proteins involved in cell division (*20*). CDK12 appears to be a multitasking kinase critical to several aspects of gene expression. Given that other CTD kinases functionally phosphorylate non-CTD substrates (*21*, *22*), CDK12 may have additional substrates contributing to the observed phenotypes upon CDK12 depletion or inhibition.

The polymerase-associated factor 1 complex (PAF1C), which is composed of subunits PAF1, LEO1, CTR9, CDC73, and RTF1 (*23*), is a crucial transcriptional regulator that acts as a multifunctional platform with broad effects on gene transcription (*24*, *25*), including processive transcription elongation (*26*, *27*). PAF1C can function as a transcription platform by facilitating the recruitment of key elongation factors, including transcription elongation factor SII (TFIIS), the “facilitates chromatin transcription” (FACT) complex, histone-modifying enzymes, and the 5,6-dichloro-1-β-D-ribofuranosylbenzimidazole (DRB) sensitivity-inducing factor (DSIF) (*28*). CDK12 has been linked with PAF1C through protein-protein interaction studies, and CDK12-catalyzed CTD phosphorylation at gene bodies has been suggested to promote the interaction of elongating Pol II with elongation and termination factors, including some components of PAF1C (LEO1 and CDC73) and SUPT6H (*13*, *19*, *29*). Decreased interaction of elongation factors PAF1C and SUPT6H with elongating Pol II caused by CDK12 inhibition has been proposed to explain the Pol II elongation defects (*13*, *27*). However, the extent to which CDK12 acts on these elongation factors and the detailed mechanisms for how it does remain unclear.

Inactivating mutations of CDK12 are associated with the progression and metastasis of a subset of ovarian, breast, and prostate cancers, which have been demonstrated to have a “BRCAness” phenotype with associated hypersensitivity to DNA damage agents and PARP1/2 [poly(adenosine diphosphate–ribose) polymerase 1/2] inhibitors (*30*–*32*). CDK12 is also overexpressed in some human epidermal growth factor receptor 2–positive breast cancers and a subset of endoplasmic reticulum–positive breast and prostate cancers (*33*). Consequently, small-molecule inhibitors targeting CDK12 are of great interest as potential targeted cancer therapies (*15*, *34*, *35*). However, the precise targets and mechanisms through which CDK12 regulates gene expression are largely undefined. We therefore hypothesized that a more systematic identification of CDK12 substrates could help elucidate the biological roles of CDK12.

Using a chemical genetic strategy for CDK12 kinase-substrate mapping by mass spectrometry (MS) (*36*, *37*), we sought to identify CDK12 substrates using conditions that preserve nuclear context and architecture. We identified 110 CDK12-specific phosphopeptides and 65 potential CDK12 substrates, which were enriched for proteins implicated in transcription, chromatin organization, and RNA splicing. The LEO1 subunit of PAF1C was validated as a bona fide CDK12 substrate both in vivo and in vitro. Mutations in LEO1 (S607, S608, and S610) to nonphosphorylatable alanine residues lead to inefficient transcription elongation in human cells and impair the association of PAF1C with elongating Pol II and chromatin. Moreover, using MS analysis, we found that LEO1 can interact with the serine/threonine phosphatase Integrator-PP2A complex (INTAC) (*38*–*41*) and that phosphorylation at S607, S608, and S610 is dephosphorylated by INTAC. Therefore, in addition to its role in RNA Pol II CTD phosphorylation, CDK12 can enhance transcription elongation through LEO1 phosphorylation, while INTAC-mediated dephosphorylation helps fine-tune processive transcription elongation.

## RESULTS

### A landscape of nuclear human CDK12 substrates identified by in situ nuclear phosphorylation

**Fig. 1.**
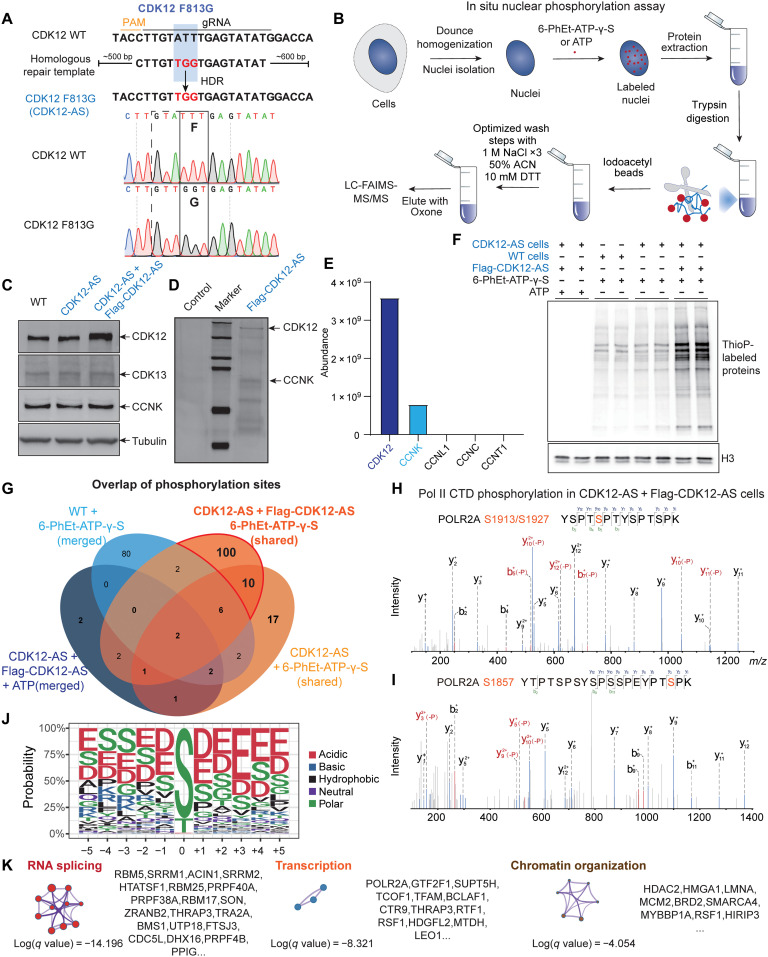
Identification of CDK12 substrates by in situ nuclear phosphorylation analysis. (**A**) Generation of HCT116 CDK12-AS cells by CRISPR-Cas9 and homology-directed repair (HDR). CDK12-AS contains the mutation of the gatekeeper F813 residue to glycine and can specifically accept bulky adenine analogs in its active site (*12*, *42*). (**B**) Schematic diagram for identifying CDK12 substrates using in situ nuclear phosphorylation assays and MS. Thiophosphopeptides were eluted with oxone and analyzed using LC-MS. (**C** to **E**) Ectopic expression of Flag-CDK12-AS in CDK12-AS cells. The expression levels of CDK12, CDK13, and CCNK were measured by immunoblotting (C). Flag-CDK12-AS was further purified by anti-M2 beads (D) and subjected to MS analysis (E). (**F**) Thiophosphorylated proteins were detected under different conditions by incubating nuclei isolated from HCT116 wild-type (WT) cells and CDK12-AS cells, with or without Flag-CDK12-AS, with 6-PhEt-ATP-γ-S or ATP as indicated. The resulting thiophosphorylated proteins were alkylated with PNBM and analyzed using immunoblotting for thiophosphate esters (ThioP). The ectopic expression of Flag-CDK12-AS enhanced labeling efficiency and provided stronger signals than the CDK12-AS group. (**G**) Venn diagram of MS-identified phosphorylation sites from four groups of samples. 6-PhEt-ATP-γ-S–labeled WT nuclei and ATP-labeled CDK12-AS nuclei with Flag-CDK12-AS served as negative controls for background subtraction. A total of 110 sites were identified as potential phosphorylation sites of CDK12-AS. Merged, merged hits from replicates; Shared, overlapped hits from replicates. (**H** and **I**) MS2 spectrum of the Pol II CTD peptide YSP TSP TYS PTS PK (H) and YTP TSP SYS PSS PEY PTS PK (I) confirmed that CDK12-AS phosphorylated Pol II CTD in the kinase assays. (**J**) CDK12 consensus recognition sequence-derived from phosphopeptides by CDK12. (**K**) Selected gene ontology terms for CDK12 substrates were enriched in the transcription, chromatin organization, and RNA splicing terms.

To identify CDK12 substrates, we developed a chemical genetic strategy using CDK12 analog-sensitive (CDK12-AS) cells and in situ nuclear phosphorylation assays. First, we used CRISPR-Cas9 and a homologous repair template to generate the HCT116 CDK12-AS cells (Fig. 1A), which contain the gatekeeper F813G mutation and can specifically accept bulky adenine analogs in its active site (*12*, *42*). We isolated nuclei using hypotonic conditions to preserve nuclear architecture (*37*) and used the isolated nuclei in conjunction with the N6-(2-phenylethyl)adenosine-5′-O-(3-thiotriphosphate) (6-PhEt-ATP-γ-S) or adenosine triphosphate (ATP) to label nuclear CDK12 substrates (Fig. 1B). The nuclei were lysed and digested with trypsin, and the resulting peptides containing either thiophosphate or cysteine were bound to the iodoacetyl beads (*36*). We optimized the wash procedures with high salt and dithiothreitol (DTT) to remove nonspecific binding and cysteine-containing peptides, respectively. The thiophosphopeptides were eluted with oxone and analyzed by liquid chromatography with tandem MS (LC-MS/MS) (Fig. 1B).

To enhance labeling efficiency, we ectopically expressed Flag-CDK12-AS in the CDK12-AS cells and confirmed the elevated expression of CDK12-AS (Fig. 1C). We further purified Flag-CDK12-AS (Fig. 1D) and performed MS analysis, which confirmed that Flag-CDK12-AS could pair correctly with CCNK in these cells (Fig. 1E). We performed in situ kinase assays using 6-PhEt-ATP-γ-S and nuclei isolated from HCT116 wild-type (WT) cells and CDK12-AS cells with or without Flag-CDK12-AS (Fig. 1F). Thiophosphorylated proteins were alkylated with *p*-nitrobenzyl mesylate (PNBM) and were analyzed by immunoblotting with anti–thiophosphate ester (ThioP) antibody. The results showed that ectopic expression of Flag-CDK12-AS could provide stronger signals than CDK12-AS cells and could increase efficiency of 6-PhEt-ATP-γ-S labeling (Fig. 1F).

Next, we performed trypsin digestion, purified thiophosphorylated peptides, and analyzed them via MS with four groups of samples (Fig. 1, F and G). We used 6-PhEt-ATP-γ-S–labeled WT nuclei and ATP-labeled CDK12-AS nuclei with ectopic expression of Flag-CDK12-AS as negative controls, as well as merged the phosphorylation sites from both replicates for background subtraction. We overlapped the phosphorylation sites between both replicates of CDK12-AS cells (shared) and identified 27 sites specifically phosphorylated by endogenous CDK12-AS (Fig. 1G). Because of the highly repetitive nature of the CTD sequence, which lacks appropriate trypsin cleavage sites for MS analysis (*43*, *44*), searching through the noncanonical CTD repeats in the CDK12-AS group did not yield any known CTD phosphopeptides. With the ectopic expression of Flag-CDK12-AS in CDK12-AS cells, we identified 110 phosphorylation sites corresponding to 65 protein hits (fig. S1A and table S1) in both biological replicates using 6-PhEt-ATP-γ-S (Fig. 1G). A search for noncanonical CTD repeats identified catalyzed Pol II CTD phosphorylation at S1913/S1927 in both replicates and S1857 in one replicate (Fig. 1, H and I), indicating that ectopic expression of Flag-CDK12-AS enhanced the labeling efficiency for substrate identification. These differences could be attributed to insufficient levels of endogenous CDK12-AS or the possibility that some of the CDK12 substrates in the nuclei may have already been phosphorylated by CDK12-AS or other kinases with endogenous ATP, which may affect in situ labeling with 6-PhEt-ATP-γ-S.

The motif analysis of peptides with specific phosphorylation in the 6-PhEt-ATP-γ-S–treated CDK12-AS group with Flag-CDK12-AS expression showed that CDK12 could phosphorylate both the serine and threonine without requiring a following proline residue. Instead, CDK12 has a preference for phosphorylating the serine or threonine in the context of aspartate and glutamate (Fig. 1J). This preference is consistent with structural findings that show that the basic surface patches surrounding the CDK12/CCNK catalytic sites may facilitate the recognition of negatively charged substrate sequences (*9*). Gene ontology analysis of the 65 potential CDK12 substrates revealed enrichment in gene transcription, chromatin organization, and RNA splicing terms (Fig. 1K). The enrichment of RNA splicing proteins in putative CDK12 substrates is consistent with previous studies that showed that CDK12 physically interacts with RNA processing factors (*11*, *17*, *18*) and regulates cotranscriptional RNA splicing and polyadenylation (*16*, *19*).

To assess the specificity of CDK12 toward these sites, we compared them with CDK9 (*22*) and CDK2 (*37*) substrates identified by analog-sensitive kinases and proteomics approaches. We found that seven proteins, including SUPT5H (S806), were exclusively shared between CDK9 and CDK12, while a similar comparison between CDK12 and CDK2 yielded six proteins in common (fig. S1B and table S2). All three kinases shared three substrate proteins, namely, LMNA, TCOF1, and SRRM2. However, most of the CDK12 phosphorylation sites were not shared with CDK9 and CDK2 at the phosphorylation site level (table S2). The minimal overlap between CDK12 and CDK9 or CDK2 is consistent with their different recognition motif sequences (Fig. 1J) and indicates that we can reliably distinguish substrates of closely related kinases with disparate functions.

### Characterization of high-confidence CDK12 substrates in cells using phosphoproteomic analysis

**Fig. 2.**
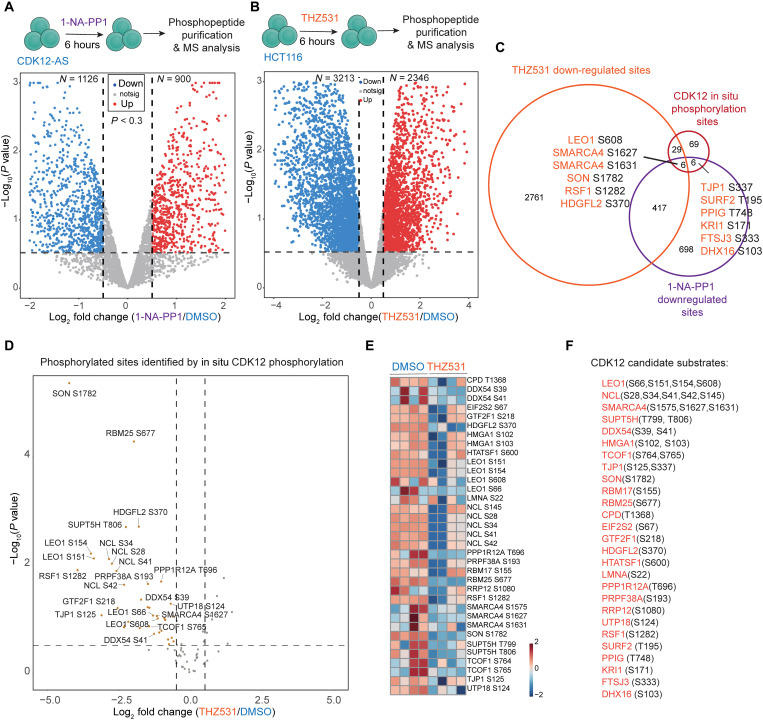
Characterization of high-confidence CDK12 substrates in cells using phosphoproteomic analysis. (**A**) Log_2_FC volcano plot of differentially phosphorylated sites in CDK12-AS cells treated with 1-NA-PP1 for 6 hours [relative to dimethyl sulfoxide (DMSO) treatment]. Highlighted points represent phosphorylation sites with a *P* value of <0.3 and Log_2_FC > 0.5 relative to DMSO treatment, and the numbers of up-regulated and down-regulated sites were labeled. (**B**) Phosphoproteomic analysis of HCT116 cells after THZ531 treatment for 6 hours. (**C**) Venn diagram depicting the overlapping of down-regulated phosphorylation sites by CDK12 inhibition or THZ531 inhibition with the 110 in situ CDK12 phosphorylation sites. (**D**) Log_2_FC volcano plots of the 110 in situ CDK12 phosphorylation sites after treatment of THZ531 for 6 hours. (**E** and **F**) Heatmap analysis of the down-regulated CDK12 phosphorylation sites by THZ531 (E). Four technical replicates were used for each group. Forty-one phosphorylation sites corresponding to 27 proteins were down-regulated by either 1-NA-PP1 or THZ531 (F).

Phosphorylation is generally regarded as a dynamic posttranslational modification critical for the regulation of biological processes, and phosphorylation dynamics can have crucial functional implications at the level of individual phosphorylation sites (*45*, *46*). To identify the CDK12 phosphorylated sites that are sensitive to CDK12 inhibition in cells, we treated CDK12-AS cells with the reversible and cell-permeable inhibitor of analog-sensitive kinases, 1-naphthyl PP1 (1-NA-PP1), for 6 hours and performed phosphopeptide purification with titanium dioxide for MS analysis (fig. S1, C to H) (*47*). We identified 1126 down-regulated [log_2_ fold change (log_2_FC) < −0.5 and *P* < 0.3] and 900 up-regulated (log_2_FC > 0.5 and *P* < 0.3) phosphorylated sites following 6 hours of selective CDK12 inhibition in CDK12-AS cells (Fig. 2A and table S3), suggesting that CDK12 inhibition dynamically regulates the phosphorylation of a variety of proteins. Because CDK12 and CDK13 are evolutionarily related and structurally similar kinases, they act substantially redundantly in Pol II CTD phosphorylation and transcription elongation (*12*). Therefore, we treated HCT116 cells with THZ531, a selective and covalent inhibitor of both CDK12 and CDK13 (*15*), for 6 hours and conducted phosphoproteomic analysis. We found that 6 hours of THZ531 treatment induced 3213 down-regulated and 2346 up-regulated phosphorylated sites (Fig. 2B and table S3), indicating that dual inhibition of CDK12/13 induced more differentially phosphorylated sites compared to CDK12 inhibition alone.

Next, we integrated directly labeled phosphorylated sites by CDK12 with the phosphoproteomic data to identify high-confidence CDK12 substrates. We overlapped the down-regulated sites by THZ531 and 1-NA-PP1 with the 110 CDK12 in situ phosphorylated sites (Fig. 2C) and found that all three groups shared only 6 phosphorylation sites including LEO1 (S608), SMARCA4 (S1627 and S1631), SON (S1782), RSF1 (S1282), and HDGFL2 (S370). Because of the redundancy between CDK12 and CDK13, criteria with shared sites among all three groups would potentially miss some CDK12 substrates. Thus, we asked which CDK12 phosphorylated sites were sensitive to 1-NA-PP1 or THZ531 treatment. We determined the changes of the 110 potential CDK12 phosphorylation sites identified by in situ phosphorylation (fig. S1A) upon 6-hour THZ531 treatment in HCT116 cells. THZ531 significantly decreased the phosphorylation of 35 CDK12 phosphorylation sites, which were related to 22 unique protein hits (Fig. 2, D and E). We combined these 35 phosphorylation sites with 6 phosphorylation sites down-regulated by 1-NA-PP1 in the CDK12-AS cells (Fig. 2C) and obtained a total of 41 phosphorylation sites (corresponding to 27 protein hits). These sites were directly phosphorylated by CDK12 and were sensitive to CDK12 or dual CDK12/13 inhibition (Fig. 2F). These 27 proteins were considered as high-confidence CDK12 substrates, which included transcription elongation factors, such as LEO1 and SUPT5H; splicing factors; and chromatin remodeling factors, suggesting multiple levels of transcriptional and cotranscriptional regulation by CDK12.

### Validation of the transcription elongation factor LEO1 as a bona fide CDK12 substrate

**Fig. 3.**
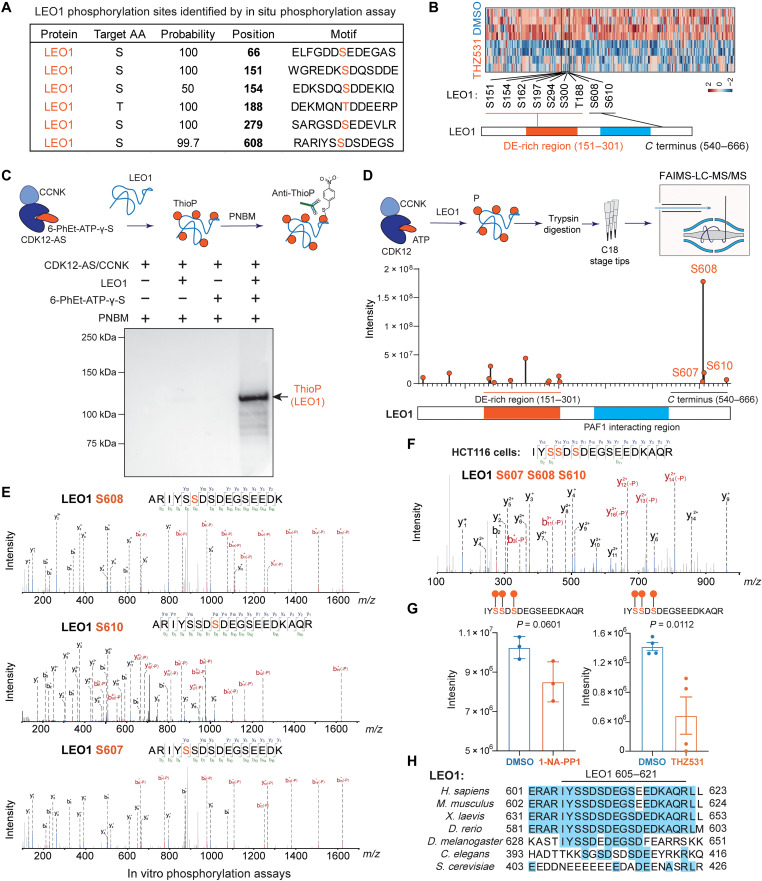
Characterization of transcription elongation factor LEO1 as a bona fide substrate of CDK12 kinase. (**A**) Multiple sites of LEO1 phosphorylated by CDK12-AS in situ. The phosphorylated sites are highlighted. AA, amino acid. (**B**) The down-regulated LEO1 phosphorylation sites by THZ531 were mainly distributed at the DE-rich and C-terminal regions. Four technical replicates were used for each group. (**C**) Detection of LEO1 thiophosphorylated by CDK12-AS. Recombinant LEO1 was incubated with CDK12-AS (kinase domain)/CCNK in the presence or absence of 6-PhEt-ATP-γ-S. After alkylation with PNBM, proteins were analyzed by immunoblotting for ThioP. (**D**) Purified full-length CDK12/CCNK was incubated with recombinant LEO1 protein in the presence of ATP for in vitro kinase assay. MS was used to characterize the LEO1 phosphorylation sites by CDK12 kinase. CDK12 mainly phosphorylate LEO1 at the DE-rich and C-terminal region, and the abundance of each phosphorylation site was depicted. (**E**) MS2 spectrum of the LEO1 phosphopeptides corresponding to individual S608, S610, and S607 in the CDK12 kinase assays. (**F**) MS2 spectrum of the LEO1 phosphopeptide IYS SDS DEG SEE DKA QR (S607P, S608P, and S610P; 3xPhospho) showing these three closely related sites were phosphorylated simultaneously in HCT116 cells. (**G**) Quantification of phosphopeptide (S607P, S608P, and S610P; 3xPhospho) after CDK12-AS inhibition or THZ531 treatment. Both CDK12-AS inhibition and THZ531 treatment for 6 hours reduce the abundance of the 3xPhospho phosphopeptide. Statistical analysis was performed with an unpaired *t* test. (**H**) Sequence alignment of LEO1 (605 to 621) from seven different species. The region of human LEO1 (605 to 621) is well conserved in vertebrates, with S607 and S608 of human LEO1 being conserved in *D. melanogaster*.

CDK12 is known to promote gene transcription elongation in collaboration with CDK13 (*12*–*14*), and these effects are typically attributed to CTD phosphorylation. However, we found that the transcription elongation factor LEO1, which is a core subunit of the global Pol II transcription regulator PAF1C, was phosphorylated at multiple sites by CDK12-AS in the nuclear phosphorylation assays (Fig. 3A). Heatmap analysis of candidate proteins revealed that multiple phosphorylation sites of LEO1 were down-regulated by a 6-hour THZ531 treatment and that these sites were mostly located within the glutamine/asparagine (DE)–rich (151 to 301) and C-terminal (540 to 666) regions (Fig. 3B). To determine whether CDK12 could directly phosphorylate LEO1 in vitro, we purified recombinant full-length LEO1 protein from *Escherichia coli* (fig. S2A) and isolated an analog-sensitive CDK12 kinase domain in complex with CCNK from human embryonic kidney (HEK) 293T cells (fig. S2B). CDK12-AS/CCNK complexes were incubated with recombinant LEO1 protein in the presence of 6-PhEt-ATP-γ-S or ATP. Immunoblotting for ThioP after alkylating the thiophosphorylated proteins with PNBM demonstrated that the CDK12-AS/CCNK complex specifically thiophosphorylated LEO1 in the presence of 6-PhEt-ATP-γ-S (Fig. 3C). Moreover, to further validate the kinase activity of CDK12 for LEO1, we purified the full-length CDK12/CCNK complex from HEK293T cells (fig. S2C) and performed ADP-Glo kinase assays using CDK12/CCNK and LEO1, which demonstrated that CDK12 can phosphorylate LEO1 in vitro while also confirming that this kinase activity was sensitive to THZ531 inhibition (fig. S2D).

The MS analysis identified the sites of in vitro phosphorylation of LEO1 by the full-length CDK12/CCNK complex, which predominantly phosphorylated LEO1 at DE-rich and C-terminal regions (Fig. 3D). S608 was found to be a hotspot for CDK12/CCNK activity (Fig. 3, D and E), and the surrounding sequence of S608 was enriched with acidic and negatively charged amino acids, consistent with CDK12 substrate motif analysis (Fig. 1J). The MS/MS spectra showed that the nearby S607 and S610 could also be phosphorylated by CDK12 (Fig. 3E). Notably, the individual phosphorylated sites of S607, S608, and S610 were found in vitro (Fig. 3E), but these sites are usually phosphorylated simultaneously in vivo as evidenced by the phosphoproteomic data from HCT116 cells (Fig. 3F), and the single and double phosphorylated peptides were not detected in HCT116 cells. Furthermore, CDK12 inhibition or THZ531 treatment significantly reduces the abundance of LEO1 phosphopeptide (605 to 620) with all three phosphorylation sites (Fig. 3G). Sequence alignment of the surrounding sequences from seven different species showed that this region is conserved from *Drosophila melanogaster* to humans (Fig. 3H), suggesting potential regulatory functions for these phosphorylation events in metazoan gene transcription.

Because CDK12 could phosphorylate LEO1 both in vitro and in vivo, we checked whether CDK12 kinase activity is required for PAF1C-mediated transcriptional regulation. Precise run-on sequencing (PRO-seq) (*48*) was performed in HCT116 CDK12-AS cells with or without 1-NA-PP1 treatment to measure the elongating Pol II. Inhibition of CDK12 activity reduced PRO-seq signals toward gene end and decreased elongating Pol II at gene bodies (fig. S2, E and F). These findings are in line with previous studies demonstrating that CDK12 kinase activity is essential for transcription elongation (*7*, *12*–*15*). In addition, we conducted Pol II, PAF1, and LEO1 chromatin immunoprecipitation sequencing with reference exogenous genome (ChIP-Rx) in CDK12-AS cells and demonstrated that 1-NA-PP1 treatment inhibited PAF1 and LEO1 occupancy at gene bodies (fig. S2, G and H). Rescaled metagene analyses of the ratio of PAF1 and LEO1 occupancies to total Pol II showed a gradual decrease in these ratios along gene bodies (fig. S2, I and J). This result also aligns with a previous study (*13*), showing that CDK12 inhibition reduces the association of PAF1C with elongating Pol II across the gene bodies.

### CDK12 phosphorylates LEO1 for processive transcription elongation

**Fig. 4.**
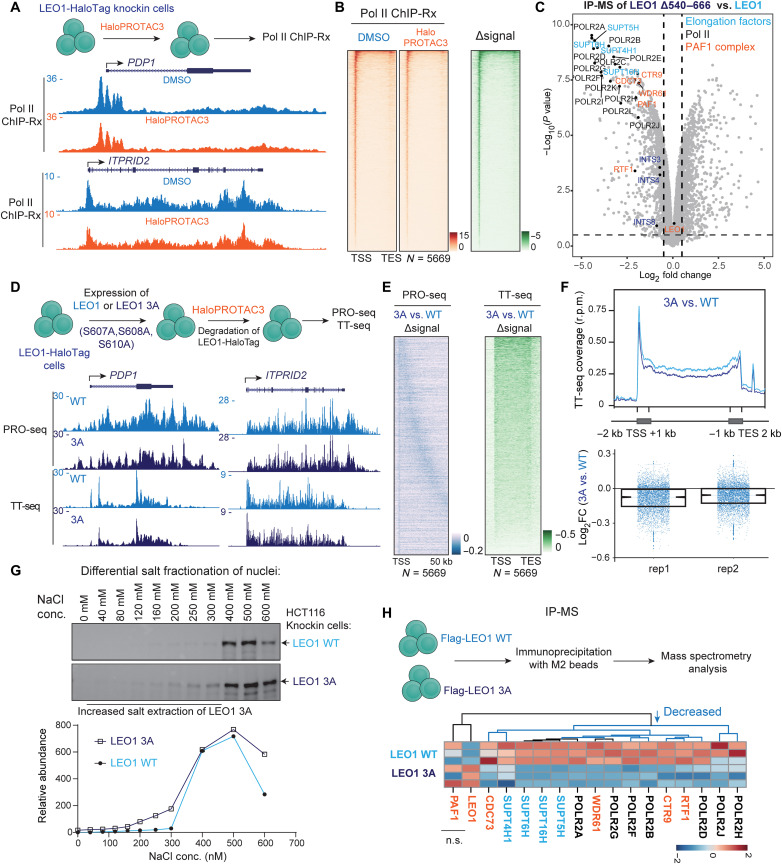
CDK12 phosphorylates LEO1 for processive transcription elongation. (**A**) Pol II ChIP-Rx analysis of LEO1-HaloTag knockin cells after DMSO or HaloPROTAC3 treatment for 6 hours. Genome browser tracks of Pol II ChIP-Rx are shown at the *PDP1* and *ITPRID2* loci. Acute degradation of LEO1 reduced Pol II occupancy at gene promoter and gene body regions. (**B**) Genome-wide analysis of Pol II ChIP-Rx at all LEO1-occupied genes (*N* = 5669) after DMSO or HaloPROTAC3 treatments. Pol II occupancy was broadly reduced at promoters and in gene bodies upon LEO1 loss. TSS, transcription start site; TES, transcription end site. (**C**) Volcano plots of LEO1 Δ540–666 versus the full-length LEO1. Deletion of LEO1 540 to 666 impairs the interaction of LEO1 with Pol II, PAF1 components, and elongation factors such as SUPT4H1, SUPT5H, SUPT6H, and SUPT16H. (**D**) Schematic diagram for investigating a triple mutation of LEO1 at S607A, S608A, and S610A (3A) on processive transcription elongation. Genome browser snapshots of PRO-seq and TT-seq at the *PDP1* and *ITPRID2* genes are shown. (**E**) Heatmap of the changes of PRO-seq and TT-seq signals between LEO1 WT and LEO1 3A mutant at the 5669 LEO1-occupied genes. LEO1 3A reduced elongating Pol II at gene bodies and the 3′ ends of genes. (**F**) Rescaled metagene plots of spike-in normalized TT-seq signals [reads per million (r.p.m.)] in LEO1 WT and LEO1 3A cells (top). Boxplot analysis of the log_2_FCs of 3A versus WT TT-seq signals is shown in the bottom for two replicates. (**G**) Differential salt fractionation of nuclei to analyze chromatin-associated LEO1 proteins. LEO1 3A knockin cells or WT cells were digested with MNase and extracted with different concentrations of salt for immunoblotting with an anti-LEO1 antibody. Quantification was performed with ImageJ. (**H**) Immunoprecipitation-MS (IP-MS) analysis of LEO1 WT and LEO1 3A using HEK293T cells. LEO1 3A shows decreased interaction with Pol II subunits and multiple elongation factors.

To investigate the contribution of CDK12-mediated LEO1 phosphorylation to efficient transcription elongation, we synthesized the HaloPROTAC3 compound incorporating a small-molecule VHL ligand (fig. S3, A and B) to degrade HaloTag fusion proteins rapidly (*49*) and generated LEO1-HaloTag knockin HCT116 cell lines using CRISPR-Cas9 editing and homologous repair (fig. S4A). HaloPROTAC3 treatment of LEO1-HaloTag knockin HCT116 cells led to rapid degradation of LEO1-HaloTag with no apparent degradation of Pol II (fig. S4B). Next, we performed Pol II ChIP-Rx in these cells after 6 hours of HaloPROTAC3 treatment (Fig. 4A). Degradation of LEO1 by HaloPROTAC3 reduced Pol II occupancy across the transcription units (Fig. 4B and fig. S4C), which was similar to the effect of THZ531 treatment on HCT116 cells (fig. S4, D and E). As CDK12 mainly phosphorylates LEO1 sites in the DE-rich region and near the C terminus of LEO1 (Fig. 3D) and these sites are sensitive to THZ531 treatment in HCT116 cells (Fig. 3B), we investigated whether these two regions are biologically relevant to CDK12’s roles in transcription elongation.

We purified full-length LEO1 and two LEO1 mutants (Δ151–301 and Δ540–666) expressed in HEK293T cells and performed immunoprecipitation-MS (IP-MS) analysis (fig. S4F). Deletion of the LEO1 C terminus (Δ540–666) impaired the interaction of LEO1 with multiple Pol II subunits, PAF1C subunits, and other elongation factors (SUPT5H, SUPT4H1, SUPT6H, and SUPT16H) compared to full-length LEO1 (Fig. 4C). This finding suggests that the LEO1 C terminus is necessary for the interaction between PAF1C and elongating Pol II complex. In contrast, internal deletion of the DE-rich region (Δ151–301) did not have similar effects (fig. S4G). Moreover, we ectopically expressed full-length LEO1 or LEO1 Δ540–666 in LEO1-HaloTag knockin HCT116 cells and used HaloPROTAC3 to induce the degradation of endogenous LEO1-HaloTag proteins (fig. S4H). Track examples and genome-wide analysis of ChIP-Rx and PRO-seq signals (fig. S5, A to C) confirmed that deletion of the LEO1 C terminus reduced Pol II at gene bodies (fig. S5D) and reduced PRO-seq signals toward the gene end (fig. S5C). This result was similar to that of LEO1 degradation (fig. S4C), suggesting that the LEO1 C terminus is required for LEO1’s function in transcriptional regulation.

To assess whether phosphorylation events of LEO1 at the C-terminally located S607, S608, and S610 sites are necessary for its role in transcription elongation, we introduced nonphosphorylatable alanine residues (3A) at these sites and conducted PRO-seq analysis (Fig. 4D), following a similar strategy as used for LEO1 Δ540–666 (fig. S5A). The loss of these three phosphorylation sites resulted in inefficient transcription elongation, indicated by reduced PRO-seq signals at gene bodies (fig. S5E) and transcription end site (TES) regions (Fig. 4, D and E). By calculating the fold changes in Pol II occupancy, we have observed that THZ531 induced a time-dependent decrease in Pol II across the gene body and at the TES regions (fig. S4E).

To confirm the effects of LEO1 3A on transcription elongation, we measured the transient transcriptome sequencing (TT-seq) (*50*), which primarily captures nascent RNA transcribed by productively elongating Pol II, in cells expressing LEO1 WT or LEO1 3A mutant. We observed a decrease in processive elongation of Pol II because of the mutation of these three sites, as evidenced by decreased TT-seq signals using heatmap, metagene, and boxplot analyses (Fig. 4, E and F). To compare the biochemical characteristics of LEO1 WT and the 3A mutant, we conducted a differential salt fractionation of nuclei from generated LEO1 3A knockin and WT cells. We found that LEO1 3A was extracted from the insoluble chromatin fractions at lower salt concentrations compared to LEO1 WT (Fig. 4G), which was further validated by comparing LEO1-HaloTag knockin HCT116 cells that expressed either LEO1 3A or LEO1 WT (fig. S5, F and G) and suggested that LEO1 3A had a weaker association with chromatin. In addition, we conducted IP-MS of LEO1 WT and LEO1 3A expressed in HEK293T cells (Fig. 4H). While the interaction with PAF1 remained largely unchanged, LEO1 3A showed reduced interactions with other PAF1C subunits (CTR9, RTF1, CDC73, and WDR61), other elongation factors (SUPT5H, SUPT4H1, SUPT6H, and SUPT16H), and Pol II. These results are consistent with our findings from LEO1 Δ540–666 IP-MS (Fig. 4C) and support our observation that inhibition of CDK12 kinase activity led to a greater reduction of PAF1C compared to Pol II across gene bodies (fig. S2, I and J).

### Identification of the INTAC as a putative phosphatase for LEO1

**Fig. 5.**
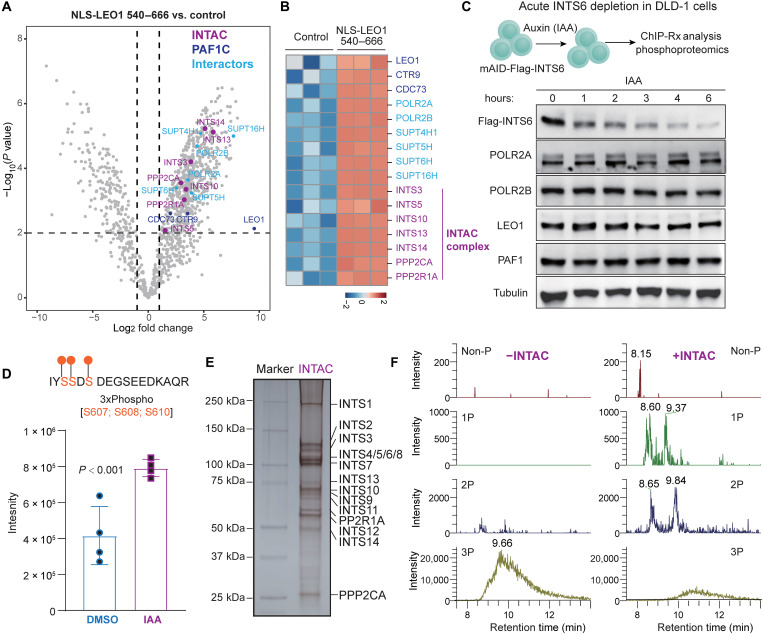
Dephosphorylation of LEO1 by the INTAC. (**A** and **B**) IP-MS analysis of Flag-tagged NLS-LEO1 C terminus (540 to 666) versus control. The volcano plot of LEO1 540 to 666 versus control shows enrichment of Pol II subunits, elongation factors, and the INTAC complex with the LEO1 C terminus (A). Multiple subunits of the INTAC complex could interact with the LEO1 C terminus (B). *Z* scores of protein abundances are plotted. (**C**) Acute INTS6 depletion in DLD-1 cells. mAID-Flag-INTS6 cells were treated with auxin (IAA) for rapid degradation of the key INTAC component INTS6. Immunoblotting for INTS6, POLR2A, POLR2B, PAF1, and LEO1 was performed, and tubulin was used as a loading control. (**D**) Phosphoproteomic analysis of mAID-Flag-INTS6 cells after DMSO or 500 μM IAA treatments for 6 hours confirms the increased detection of the LEO1 phosphopeptide IYS SDS DEG SEE DKA QR (S607P, S608P, and S610P; 3xPhospho) upon acute INTAC loss. (**E**) Silver staining of human INTAC complex purified from HEK Expi293 cells (*38*). (**F**) INTAC dephosphorylates LEO1 peptides in vitro. A synthetic peptide containing the S607P, S608P, and S610P (IYS SDS DEG SEE DC) was incubated with or without INTAC. After the phosphatase reaction, the peptides were desalted with C18 StageTips for ESI-MS analysis. Chromatograms of non-P, 1P, 2P, and 3P peptides in phosphatase products with or without INTAC. INTAC could dephosphorylate the 3P peptide to non-P, 1P, and 2P peptides. Multiple peaks on the chromatograms represent the isomers of 1P and 2P peptides.

Sequence alignments of the LEO1 C terminus showed strong conservation among vertebrates (fig. S5H), indicating the importance of this region in LEO1 function. To investigate further, we purified the LEO1 C terminus (540 to 666) with an N-terminal nuclear localization sequence, allowing for proper nuclear distribution, and performed IP-MS analysis. We found that the LEO1 C terminus could interact with Pol II subunits (POLR2A and POLR2B), DSIF subunits SUPT5H and SUPT4H1, PAF1C subunits CTR9 and CDC73, and FACT subunit SUPT16H (Fig. 5A), suggesting that the LEO1 C terminus may function as a platform for interactions between Pol II and other elongation factors that promote transcription elongation. We also observed that the LEO1 C terminus interacted with the noncanonical INTAC (*38*–*41*). Multiple subunits of INTAC, including the Integrator components INTS3, INTS5, INTS10, INTS13, and INTS14 as well as the serine-threonine protein phosphatase PP2A core enzyme (PPP2R1A and PPP2CA) were specifically enriched in the IP-MS analysis of LEO1 C terminus interactors (Fig. 5B), raising the possibility that INTAC modulates LEO1 function.

Thus, we performed auxin-inducible acute depletion of INTS6, a subunit that bridges the interaction between Integrator and PP2A (*38*, *40*), in the mAID-Flag-INTS6 DLD-1 cells (*38*). Immunoblotting of Flag-INTS6 confirmed the time-dependent acute depletion of INTS6 and showed that it did not affect the total protein levels of Pol II subunits (POLR2A and POLR2B) or PAF1 subunits (PAF1 and LEO1) (Fig. 5C). We induced the acute depletion of INTS6 in the mAID-Flag-INTS6 cells for 6 hours and performed phosphoproteomic analyses. Rapid degradation of INTS6 significantly increased the abundance of the phosphorylated peptides containing S607, S608, and S610 phosphorylation (Fig. 5D). To determine whether INTAC can directly dephosphorylate LEO1, we performed a phosphatase assay using purified INTAC (Fig. 5E) (*38*) and a synthetic peptide triply phosphorylated at S607, S608, and S610 (3P). Electrospray ionization–MS (ESI-MS) analysis showed that INTAC can directly dephosphorylate LEO1 at S607, S608, and S610 in vitro (Fig. 5F). Together, these results demonstrate that INTAC acts as a putative phosphatase for LEO1 phosphorylation at these sites.

### INTAC and CDK12 fine-tune the interaction between PAF1C and Pol II

**Fig. 6.**
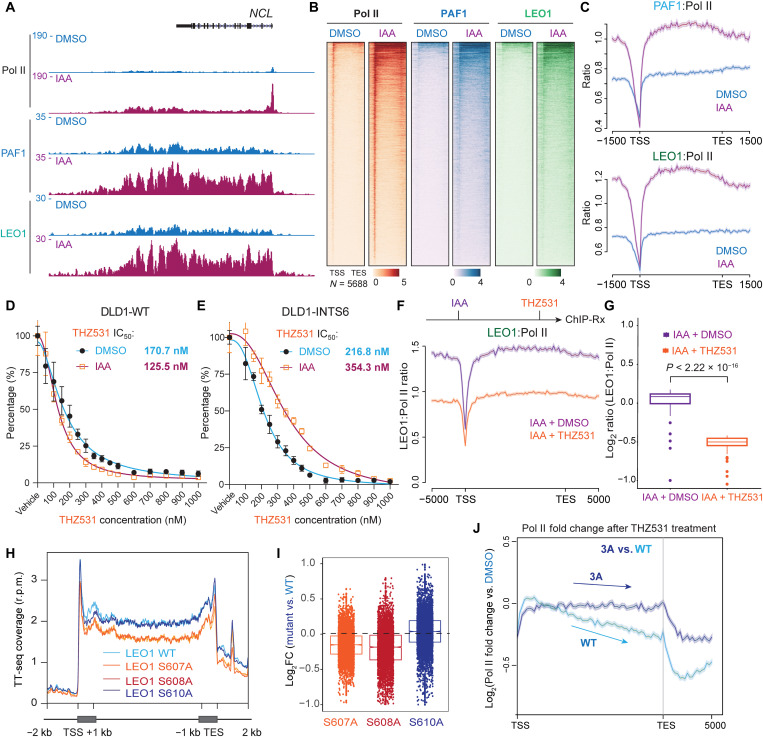
Antagonism between THZ531 and acute INTS6 depletion on PAF1C and Pol II interaction. (**A**) ChIP-Rx track examples of Pol II, PAF1, and LEO1 in mAID-Flag-INTS6 cells treated with either DMSO or IAA. (**B**) Heatmaps of Pol II, PAF1, and LEO1 ChIP-Rx signals at 5688 LEO1-occupied genes in mAID-Flag-INTS6 cells after DMSO or 500 μM IAA treatments for 6 hours. (**C**) Meta-analyses of rescaled LEO1-occupied genes showing ratios of PAF1 (top) or LEO1 (bottom) to Pol II coverage (r.p.m.). Acute loss of INTAC increases the ratio of PAF1C versus Pol II across the gene body to the TES region. (**D** and **E**) Dose-dependent inhibitory effects of THZ531 in DLD-1 WT and mAID-Flag-INTS6 cells in the absence or presence of IAA. After treatments, cells were cultured for 5 days and were measured by the CellTiter-Glo luminescent cell viability assays (*n* = 4). Median inhibitory concentration (IC_50_) values were calculated by nonlinear regression with four parameters. (**F** and **G**) THZ531 treatment further decreased the LEO1:Pol II ratio elevated by INTS6 depletion. The mAID-Flag-INTS6 cells were treated with IAA for 5 hours and further treated with DMSO or 500 nM THZ531 for another hour before LEO1 and Pol II ChIP analysis. Metagene analyses of rescaled LEO1-occupied genes showing ratios of LEO1 to Pol II coverage (r.p.m.) with or without further THZ531 treatment. Quantification of the LEO1:Pol II ratios was plotted in the boxplots (G). (**H** and **I**) TT-seq analysis in HCT116 cells with LEO1 single-point mutations (H). Mutations of either LEO1 S607 or S608 (but not S610) to alanine can reduce spike-in normalized TT-seq signals at gene bodies (I). (**J**) Changes of Pol II ratios after THZ531 treatment for 45 min in LEO1 WT and 3A cells. Pol II ratios were calculated by dividing the THZ531-treated Pol II occupancy to vehicle-treated Pol II signals.

Next, we conducted ChIP-Rx of Pol II, PAF1, and LEO1 to examine the effects of acute INTS6 loss in DLD-1 cells. The results revealed an increase in PAF1 and LEO1 occupancy at gene body regions, as demonstrated by track examples and heatmap analysis (Fig. 6, A and B). In addition, we calculated the ratios of PAF1 and LEO1 versus Pol II coverage [reads per million (r.p.m.)] and found that acute loss of INTS6 increased the ratios at gene bodies and TES regions (Fig. 6C), demonstrating that inhibition of INTAC led to enhanced association between PAF1C and Pol II on chromatin.

Given the opposite effects of CDK12 and INTAC on PAF1C occupancy at gene bodies, we sought to determine whether the loss of INTAC exhibited antagonism to CDK12/CDK13 inhibition. To achieve this, we induced acute depletion of INTS6 in DLD-1 cells and measured the median inhibitory concentration (IC_50_) values of THZ531 in these cells (Fig. 6, D and E). For the control WT DLD-1 cells, the treatment of THZ531 inhibited cell proliferation in a dose-dependent manner and exhibited IC_50_ values of 170.7 and 125.5 nM in the absence or presence of indole-3-acetic acid (IAA), respectively. Although a previous study (*40*) suggested that INTS6 loss did not provide a competitive growth advantage with THZ531 treatment at a single dose of 50 nM, we found that mAID-Flag-INTS6 DLD-1 cells showed the IC_50_ values of 216.8 and 354.3 nM in the absence or presence of 500 μM IAA (Fig. 6, D and E), showing that the loss of the integrator component INTS6 conferred resistance to CDK12/13 inhibition. To directly assess the antagonism of CDK12/13 inhibition to INTS6 loss on the genomic interaction between PAF1C and Pol II, we first treated mAID-Flag-INTS6 DLD-1 cells with 500 μM IAA for 5 hours to induce acute INTS6 loss and then used THZ531 to treat these cells for another hour. We found that the treatment of INTS6-depleted cells with THZ531 reduced the ratio of LEO1 to Pol II across gene bodies and at the TES regions (Fig. 6, F and G), suggesting that THZ531 exerts an antagonistic effect on INTAC loss.

To investigate the effects of LEO1 phosphorylation on transcription elongation, we also generated HCT116 knockin cell lines with individual single-point mutation (S607A, S608A, and S610A) and conducted TT-seq analysis (Fig. 6, H and I). Our findings showed that mutations of either S607 or S608 (but not S610) to alanine could reduce the TT-seq signal at gene bodies, highlighting the significance of these two residues for LEO1-mediated transcription elongation. Furthermore, to measure whether these three phosphorylation sites are involved in THZ531-mediated transcriptional inhibition, we treated HCT116 3A knockin and WT cells with THZ531 for 45 min before Pol II ChIP-Rx analysis. We compared the differences between HCT116 WT and LEO1 3A and found that the LEO1 3A mutant showed less reduction of Pol II at the 3′ end of genes and TES regions, indicating that LEO1 3A mutations reduced the sensitivity of THZ531 on the reduction of Pol II occupancy across the gene bodies and at the TES regions (Fig. 6J). Together, these findings indicate that the interplay between INTAC and CDK12 may fine-tune the phosphorylation of LEO1 for the control of gene expression.

## DISCUSSION

CDK12 has demonstrated roles in transcription elongation, cotranscriptional mRNA splicing, and DNA damage and repair. Dysfunction of CDK12 is associated with the progression and metastasis of a subset of cancers, which affects response to antineoplastic agents. Consequently, small-molecule inhibitors targeting CDK12 are of great interest for cancer treatments as potential targeted therapies (*32*, *51*). Although CDK12 has been identified as a kinase for Pol II CTD, as well as for 4E-BP1 (*20*), whether these cellular functions fully explain the essential roles of CDK12 remains unknown. We therefore developed a chemical genetic strategy to identify physiological substrates of CDK12 (Fig. 1). Building upon phosphoproteomic studies, we identified 27 previously undiscovered candidate substrates, suggesting that in situ assays may provide enhanced sensitivity for identifying substrates of CDKs and other nuclear kinases. Accordingly, these methods are readily adaptable for the study of other nuclear kinases that are amenable to analog-sensitive mutations. The 27 candidate substrates identified in this study include proteins covering a broad spectrum of nuclear metabolism, consistent with previously reported roles of CDK12 in transcriptional regulation, RNA processing (*11*, *17*, *18*), and chromatin modification (*52*, *53*).

Comparison of CDK12 substrate with published CDK9 and CDK2 substrates (*22*, *37*) revealed that only a small fraction of the CDK12 substrate set could be phosphorylated by CDK9 or CDK2 (fig. S1B and table S2). However, upon examining the individual phosphorylation sites, it was found that most CDK12 phosphorylation sites were not shared with CDK9 and CDK2, with the exception of three sites including PPP1CC T311 (shared between CDK9 and CDK12), LMNA S22 (shared among CDK9, CDK12, and CDK2), and SUPT5H T806 (a validated CDK9 substrate shared between CDK9 and CDK12) (*22*, *54*). Both LMNA S22 and SUPT5H T806 were sensitive to THZ531 treatment in HCT116 cells (Fig. 2E), and it is possible that they are shared substrates between CDK9 and CDK12. A study reported that the purified CDK9/CCNT1 complex phosphorylated reconstituted paused Pol II complexes in vitro at 49 phosphorylation sites corresponding to SUPT5H, SUPT6H, PAF1, LEO1 (S277, S300, S608, and S300), CDC73, CTR9, NELFA, and NELFE (*23*). However, a screening of CDK9 substrates using CDK9-AS and proteomics strategy did not identify LEO1 as a potential CDK9 substrate (*22*), Therefore, further investigation is required to determine whether LEO1 is a potential substrate of CDK9 in cells.

Motif analysis of CDK12 substrate peptides shows that CDK12 phosphorylates both serine and threonine residues without requiring a proximal C-terminal proline residue or an arginine or lysine at the +2 position, suggesting that CDK12 is not a typical proline-directed kinase exemplified by CDK9, CDK1, and CDK2 (*22*, *37*). Instead, CDK12 prefers to phosphorylate substrate peptides with surrounding sequences enriched in the negatively charged amino acids aspartate and glutamate (Fig. 1J). Our findings are in agreement with structural differences revealed in the CDK2/CCNA2 and CDK12/CCNK crystal structures (*8*, *9*), with the CDK12 catalytic pocket consisting of basic surface patches that can accommodate negatively charged substrate sequences. Moreover, the CDK12 and CDK13 kinase active centers were associated with a C-terminal HE motif and a polybasic cluster, which have been reported to mediate their preference for negatively charged and prephosphorylated CTD (*8*, *9*). Thus, it seems reasonable that the HE motif and polybasic cluster may contribute at least in part to the preference for negatively charged substrate sequences.

In this study, we identified LEO1 as a bona fide cellular substrate of CDK12 both in vitro and in vivo (Fig. 2). Biochemical studies revealed that the LEO1 C terminus (540 to 666) is required for the interaction of LEO1 with elongating Pol II (Fig. 4C) and that deletion of the C terminus globally impairs transcription elongation (fig. S5). The C terminus is well conserved across species (fig. S5H) and coimmunoprecipitates with Pol II, as well as elongation factors, including PAF1C subunits (Fig. 5A), suggesting that the C terminus serves as a platform for interactions between PAF1C, Pol II, and other elongation factors, leading to the promotion of gene transcription beyond previously reported roles in CTD phosphorylation (*13*, *27*). However, although the structure of human elongating Pol II complexes with a LEO1 truncation (370 to 518) shows the contacts of LEO1 with DSIF, RTF1, Pol II, and upstream DNA to stabilize the elongating Pol II complex (*23*, *26*), the C terminus of LEO1 (540 to 666) is very flexible and the exact and complete structural information regarding this region is still missing. Furthermore, we showed that the C-terminally located S607, S608, and S610 residues are essential for the association of LEO1 with elongating Pol II and chromatin, while mutation of these sites to the nonphosphorylatable alanine leads to impaired transcription elongation and an overall decrease in nascent transcription (Fig. 4). These findings suggest that modulation of the phosphorylation at these closely related and sequence-conserved sites is a key mechanism by which the LEO1 C terminus contributes to transcription elongation, although the precise mechanism for these effects warrants further investigation. Although we attempted to generate antibodies against a triply phosphorylated peptide, our immunization of eight rabbits with keyhole limpet hemocyanin-conjugated phosphorylated peptides showed specificity for the synthetic triply phosphorylated peptide, but failed to show notable affinity in ChIP sequencing (ChIP-seq) assays and immunoblotting of cell lysates. Moreover, although we demonstrated that the phosphorylation sites at LEO1 C terminus were essential for transcription elongation, we cannot at this time exclude the potential importance of other LEO1 phosphorylation sites in this process or for other cellular functions of PAF1C.

INTAC is a large and multitasking complex targeting the Pol II CTD and SUPT5H for counteracting Pol II phosphorylation and elongation (*38*, *40*, *55*, *56*). Knockout of INST6 in THP-1 cells and phosphoproteomic analysis identified a large number of potential substrates including POLR2A, SUPT5H, SUPT6H, MYC, NELFE, and LEO1, which mediate the balance between INTAC and CDK9 kinase for transcriptional regulation (*40*). Previous studies (*28*, *57*) have reported that PAF1C could facilitate the recruitment of INTAC to protein-coding genes. Our finding that the C terminus of LEO1 interacts with multiple INTAC subunits (Fig. 5, A and B) provides a biochemical explanation for the recruitment of INTAC by PAF1C and reveals an interplay or “balance” between CDK12 and INTAC on the phosphorylation of the LEO1 C terminus for fine-tuning transcription elongation.

In summary, an unbiased chemical genetic search for CDK12 substrates led to the identification, both biochemically and genetically, of a pathway that regulates transcription elongation. CDK12 inhibition or preventing site-specific phosphorylation of LEO1 by mutation led to decreased transcription elongation genome wide, while INTAC-mediated dephosphorylation of LEO1 affected the occupancy of PAF1C on chromatin. Our results not only provide a mechanism for CDK12-mediated transcription elongation beyond its role in CTD phosphorylation but also reveal that CDK12 and INTAC modulate LEO1 phosphorylation to regulate PAF1C–Pol II interaction and transcription elongation. However, it will be particularly important to decipher additional mechanisms underlying this transcriptional regulation, including how their substrates, which are not limited to LEO1, contribute to the orchestration of proper gene expression and how disruptions in this process contribute to CDK12-relevant cancers.

### Limitations of the study

This research was primarily carried out in HCT116 and DLD-1 colon cancer cell lines, and while the basic functions of LEO1 and CDK12 in transcription are likely to be conserved, the effects of LEO1 mutations or CDK12 inhibition on target genes may differ in other cell types. To identify CDK12 substrates, the study used CDK12-AS cells with ectopically expressed Flag-CDK12-AS because endogenous CDK12-AS alone was not sufficient for complete substrate identification (Fig. 1, F and G). However, it is possible that some CDK12 substrates may have already been phosphorylated by CDK12-AS or other kinases using endogenous nucleoside triphosphate, leading to the possible omission of some potential CDK12 substrates. Moreover, the study provides evidence that the LEO1 C terminus interacts with multiple elongation factors, which may affect the effect of PAF1C on Pol II elongation, but the precise molecular mechanisms require further elucidation. Last, although the study shows that CDK12 and INTAC regulate LEO1 phosphorylation, the extent and mechanism by which this phosphorylation influences genome-wide transcriptional regulation of CDK12 and INTAC require additional research.

## MATERIALS AND METHODS

### Cell culture conditions and DNA construction

HEK293T [American Type Culture Collection (ATCC), CRL-3216] and HCT116 (ATCC, CCL-247) cells were cultured in Dulbecco’s modified Eagle’s medium (DMEM) supplemented with 10% fetal bovine serum (FBS; catalog no. S711-001, LONSERA). HCT116 analog-sensitive CDK12 (CDK12-AS; F813G), HCT116-3A, and LEO1-HaloTag knockin cell lines were generated by CRISPR-Cas9–mediated homology-directed repair. DLD-1 INTS6-dTag cells (*38*) were provided by F. X. Chen of Fudan University and were maintained in DMEM complete medium. *Drosophila* S2 cells were obtained from Invitrogen (catalog no. R690-07) and maintained in Schneider’s medium (Thermo Fisher Scientific, catalog no. 21720024). Mouse embryonic fibroblast (MEF) cells (3T3 MEFs WT) were grown in DMEM with 10% FBS. All cells were cultured at 37°C with 5% CO_2_. Live cells were quantified using a TC20 automated cell counter (Bio-Rad). The cells were routinely tested for mycoplasma contamination with the mycoblue mycoplasma detector (Vazyme).

Full-length LEO1 containing an N-terminal His_10_ tag was cloned into the pET16b vector (pET16b-His-LEO1). Full-length CDK12 was cloned into the pCDH vector to generate the pCDH-3xFlag-TEV-CDK12 plasmid, which includes an N-terminal 3xFlag tag followed by a TEV (tobacco etch virus protease) cleavage site. CDK12-AS (696 to 1082) encoding the kinase domain of CDK12 (residues 696 to 1082) with a point mutation of F813G was inserted into the pCDH vector with a 3xFlag tag and a TEV recognition site [pCDH-FLAG-TEV-CDK12 (696 to 1082; F813G)]. The CCNK truncate containing the cyclin box domain (residues 1 to 267) was cloned into the pCDH-EF1 vector (Addgene, #72266) to make the pCDH-CCNK (1 to 267) plasmids. The pCDH-3xFlag-LEO1, pCDH-3xFlag-LEO1-△540-666, pCDH-3xFlag-LEO1-△151-301, pCDH-3xFlag-LEO1-3A (S607A, S608A, and S610A), and pCDH-3xFlag-NLS-LEO1-540-666 plasmids were generated to express LEO1 and LEO1 mutants in mammalian cells. CRISPR plasmids targeting CDK12, CDK13, and LEO1 were generated by the insertion of synthesized DNA into the pX330 plasmid. Donor templates containing the desired insertions or modifications, flanked by segments of DNA homologous to the blunt ends of the cleaved DNA, were cloned into the pMK286 (Addgene, #72824) or pFENHK plasmids (pFN205K HaloTag EF1a-neo Flexi). The sequences for single guide RNA, donor templates, and expression vectors were shown in table S4.

### Antibodies, peptides, and chemicals

LEO1 polyclonal antibody (catalog no. 12281-1-AP) and PAF1 polyclonal antibody (catalog no. 15441-1-A) were purchased from Proteintech. Horseradish peroxidase (HRP)–conjugated mouse anti-DDDDK-Tag monoclonal antibody (mAb) (catalog no. AE024), PAF1 rabbit mAb (catalog no. A3437), histone H3 polyclonal antibody (catalog no. A2348), and CCNK polyclonal antibody (catalog no. A10261) were purchased from ABclonal. Pol II Rpb1 NTD (D8L4Y) rabbit mAb (anti-POLR2A, catalog no. 14958) and anti-ThioP antibody (catalog no. ab92570) were obtained from Abcam. Monoclonal anti-FLAG M2 antibody (catalog no. F3165) and anti–β-tubulin (C66) mAb (catalog no. M20005) were purchased from Sigma-Aldrich and Abmart, respectively. Homemade rabbit anti-POLR2B antibody was generated as previously described (*58*). Rabbit anti-HaloTag antibody was generated in rabbits using HaloTag recombinant protein, and rabbit anti-CDK12 and anti-CDK13 antibodies were generated using recombinant CDK12 and CDK13 C-terminal protein truncates (275 and 278 amino acids), respectively. LEO1 peptide (605 to 618) IYS SDS DEG SEE DC and phosphorylated peptide IYS(P) S(P)DS(P) DEG SEE DC were synthesized and purified (purity >90%) by ChinaPeptides with further trifluoroacetic acid (TFA) removal. Recombinant INTAC complex (*38*) was provided by Y. Xu and F. X. Chen of Fudan University. 6-PhEt-ATP-γ-S (catalog no. 944834-43-9) and auxin (catalog no. 87-51-4) were purchased from BOC Sciences. 4-Thiouridine (catalog no. T4509), MTSEA-biotin-XX (catalog no. M9938), and Oxone (catalog no. 228036) were purchased from Sigma-Aldrich. CDK12 inhibitor THZ531 (catalog no. T4293) was obtained from TargetMOL and reconstituted in dimethyl sulfoxide (DMSO). ATP-γ-S (catalog no. B7582) was provided by APExBIO. DTT (catalog no. R0861) and iodoacetamide (catalog no. A39271) were provided by Thermo Fisher Scientific. PNBM (catalog no. 39628-94-9) was obtained from Abcam. 1-NA-PP1 (catalog no. 10954) was purchased from Cayman Chemical and was reconstituted in DMSO.

### Protein purification

Recombinant LEO1 protein was purified from *E. coli* BL21 strain [New England Biolabs (NEB), catalog no. C2530H] using pET16b-His-LEO1 and Ni NTA 6FF beads (Smart Lifesciences, catalog no. SA005C). The BL21 cells were induced by 0.2 mM isopropyl-β-d-thiogalactopyranoside for 16 hours at 16°C. Recombinant LEO1 protein was purified with Ni NTA 6FF beads (Smart Lifesciences, catalog no. SA005C). To purify the CDK12/CCNK complex, pCDH-3xFlag-TEV-CDK12 and pCDH-CCNK plasmids were cotransfected into HEK293T cells using polyethylenimine (Polysciences, catalog no. 23966). CDK12/CCNK complex was purified with anti-DYKDDDDK affinity beads (Smart Lifesciences, catalog no. SA042005) and eluted with TEV digestion. Analog-sensitive CDK12 (696 to 1082; F813G)/CCNK (1 to 267) complex was purified using similar strategies. INTAC complex was purified as previously described (*38*).

### Generation of HCT116 CDK12-AS (F813G), LEO1-HaloTag knockin, and LEO1 3A cells

To create analog-sensitive HCT116 cell lines, we replaced endogenous CDK12 with its analog-sensitive version via CRISPR-Cas9–mediated site-directed homologous repair. Guide RNAs targeting the gatekeeper residue of CDK12 (F813) (*18*) and repair templates harboring the analog-sensitive mutations F813G were used to create HCT116-AS cells. HCT116 LEO1–mutated cells were generated using similar methods. The edited cells were sorted as single cells using a BD FACSAria III sorter and seeded into 96-well plates. Knockin clones were verified via polymerase chain reaction and Sanger sequencing. HCT116 CDK12-AS cells were treated with 1-NA-PP1 for 6 hours and used for phosphoproteomic, ChIP-Rx, and PRO-seq analysis. LEO1 knockin clones were further confirmed by immunoblotting with the anti-HaloTag and anti-LEO1 antibodies. LEO1-HaloTag protein degradation was performed with HaloPROTAC3 at indicated concentrations and periods, before confirmation by immunoblotting.

### In situ nuclear phosphorylation assay for proteomic analysis

Two million HCT116 cells were harvested and rinsed with cold phosphate-buffered saline (PBS) and then washed twice with 10 ml of cold hypotonic lysis buffer 1 [10 mM Hepes (pH 7.4), 10 mM KCl, 2 mM MgCl_2_, and 1× proteinase inhibitors]. Cells were resuspended in 10 ml of hypotonic lysis buffer 1 for 15 min on ice and Dounce homogenized with 20 strokes using the tight pestle, followed by centrifugation at 1300*g* for 10 min. The cell pellets were resuspended with 3 ml of hypotonic lysis buffer 1 and homogenized using a 1-ml syringe with a 26-gauge needle. The slurry was added slowly to the surface of 10 ml of cold hypotonic lysis buffer 1 containing 30% sucrose (w/v). After centrifugation at 1000*g* for 10 min, the pellets were washed three times with 5 ml of hypotonic lysis buffer 1 and resuspended in 2 ml of hypotonic lysis buffer 1. For the in vitro nuclear phosphorylation assays, the nuclei were incubated with 0.5 mM MnCl_2_ and 100 μM ATP or 6-PhEt-ATP-γ-S (BOC Sciences, catalog no. 944834-43-9) at 30°C for 2 hours with gentle rotation. The nuclei were centrifuged at 3000 rpm for 15 s, and the supernatants were discarded. To purify the thiophosphorylated peptides, the nuclei were resuspended with 0.4 ml of hypotonic lysis buffer 2 [30 mM Hepes (pH 7.4), 10 mM EDTA, and benzonase (25 U/ml)]. After incubation on ice for 30 min, Tween 20 was added to a final concentration of 0.1%, and the nuclei were sonicated at 4°C with a Bioruptor plus (four cycles of 5 min at maximum output; 30-s on/30-s off). After centrifugation at 20,000*g* for 10 min, the supernatants were collected, and protein concentrations were determined by the bicinchoninic acid (BCA) assay. One milligram of protein was precipitated with acetone and washed once with −20°C acetone. Precipitates were dissolved in 150 μl of UA buffer [100 mM tris-HCl (pH 8.5) and 8 M urea] at room temperature and then diluted with 1050 μl of 50 mM tris-HCl (pH 8.0).

Proteins were digested with trypsin (Promega) overnight at 37°C with shaking (1500 rpm). The digested peptides were incubated with high-affinity iodoacetyl resin (GenScript, catalog no. L00403) on a shaker at room temperature for 5 hours. The resin was then washed once with 0.5 ml of water, three times with 0.5 ml of 1 M NaCl, once with 0.5 ml of 50% acetonitrile, and once with 0.5 ml of 10 mM DTT to remove the contaminant peptides. The sulfhydryl-containing peptides were eluted with 100 μl of oxone (1 mg/ml; pH 3.5) (Sigma-Aldrich, catalog no. 228036) at room temperature with rotation for 10 min. These eluates were further desalted by C18 StageTips (Thermo Fisher Scientific, catalog no. 87782) and used for MS analysis.

### Phosphoproteomic analysis

Phosphoproteomic analysis was performed with the EasyPhos platform as previously described (*47*). Briefly, 7 × 10^6^ cells per treatment condition were rinsed with tris-buffered saline (TBS) buffer [50 mM tris-HCl (pH 7.6) and 150 mM NaCl] and lysed with 1 ml of sodium deoxycholate (SDC) lysis buffer [4% (w/v) sodium deoxycholate and 100 mM tris-HCl (pH 8.5)]. After being heated immediately at 95°C for 5 min, the lysates were homogenized by sonication at 4°C, and the protein concentration was determined by BCA assay. One milligram of protein starting materials was reduced and alkylated by 20 mM DTT and 40 mM iodoacetamide at 37°C for 30 min in the dark and then digested with trypsin overnight at 37°C with shaking (1500 rpm). For phosphopeptide enrichment, the digested peptides were incubated with titanium dioxide (TiO_2_) beads (GL Sciences, catalog no. 5010-21315) at 40°C with shaking for 20 min, then washed five times with wash buffer (5% TFA and 60% isopropyl alcohol), and further eluted with 50 μl of freshly prepared EP elution buffer [ammonia solution:40% (v/v) acetonitrile, 1:4]. Eluted phosphopeptides were concentrated immediately using an evaporative concentrator for 30 min at 45°C. Phosphopeptides were acidified in 1% TFA and desalted with the graphite spin columns (Thermo Fisher Scientific, catalog no. 88302). Phosphopeptides were washed with 1% TFA and eluted with elution buffer (0.1% formic acid in 50% acetonitrile). These elutes were concentrated by an evaporative concentrator and reconstituted in 10 μl of loading buffer (0.2% formic acid and 2% acetonitrile).

Phosphopeptides were analyzed using an Orbitrap Exploris 480 mass spectrometer equipped with the FAIMS Pro interface. Samples were analyzed on an EASY-nLC system using a Hypersil GOLD C18 Selectivity HPLC column and a 3-hour preprogrammed gradient. Full MS resolutions were set to 60,000 at mass/charge ratio (*m*/*z*) of 200, and the mass range was set to 350 to 1500. Raw files were processed with Proteome Discoverer 2.4 with variable modifications of oxidation, acetylation (N-term), and phosphorylation (STY) allowed. To validate peptide identification, the automatic mode that controls the peptide level error rate if possible was selected. The strict target false discovery rate for peptide spectrum matches (PSMs) or peptides was set at 0.01, and peptides with lower confidence than 0.01 were excluded from the final result. The abundance of phosphorylation sites was quantified by MaxQuant.

### CDK12 kinase assay

For CDK12-AS kinase assay with 1 mM 6-PhEt-ATP-γ-S as the phosphate group donor, the recombinant CDK12-AS (696 to 1082; F813G)/CCNK (1 to 267) was incubated with 2 μg of LEO1 for 30 min at 37°C in 20 μl of kinase buffer [20 mM Hepes (pH 7.4), 150 mM NaCl, and 10 mM MgCl_2_]. Reactions were stopped by the addition of 20 mM EDTA. The products were alkylated by adding 2.5 mM PNBM (Abcam, catalog no. 39628-94-9) at room temperature with rotation for 1 hour. The thiophosphorylated proteins were investigated using immunoblotting with an anti-ThioP antibody (Abcam, catalog no. ab92570). Two micrograms of recombinant LEO1 proteins was incubated with full-length CDK12/CCNK complex in 20 μl of kinase buffer [20 mM Hepes (pH 7.4), 150 mM NaCl, 10 mM MgCl_2_, and 1 mM ATP] in the presence or absence of THZ531 (TargetMOL, catalog no. T4293). The reaction was performed at 37°C for 30 min and stopped by the addition of 20 mM EDTA. The products were used for either ADP-Glo kinase assay (Promega, catalog no. V6930) or MS analysis after trypsin digestion.

### In vitro phosphatase assay

Five micrograms of phosphorylated LEO1 peptide [IYS(p)S(p)DS(p)DEGSEEDC] was incubated with or without INTAC complex in a final volume of 100 μl of phosphatase reaction buffer containing 50 mM Hepes (pH 7.4), 100 mM NaCl, 10 mM MgCl_2_, 1 mM MnCl_2_, and 2 mM DTT. The reaction was performed at 30°C overnight and stopped by adding 1% TFA. The peptides were desalted with C18 StageTips and analyzed by liquid chromatography with a Thermo TSQ Quantis triple-stage quadrupole mass spectrometer under the positive ion mode. Injections were automatically performed using an UltiMate 3000 HPLC equipped with an autosampler. A Hypersil GOLD C18 HPLC column was used for chromatographic separation at 25°C. The electrospray ionization was set at 3.5 kV, and the vaporizer temperature was set at 275°C. Selected reaction monitoring mode was used, and the cycle time was 0.3 s. The mobile phase was composed of 0.1% formic acid H_2_O (A) and acetonitrile (B). The following gradient conditions were used: 0 to 0.5 min, 0 to 5% B; 0.5 to 18 min, 5 to 30% B; 18 to 18.1 min, 30 to 100% B; 18.1 to 19 min, 100% B; 19.0 to 19.1 min, 100 to 5% B; and 19.1 to 20 min, 5% B. The flow rate was 0.2 ml/min. The precursor *m*/*z* (+2 charges) for nonphosphorylated peptides was 768, and the product *m*/*z* was 707.72 and 759.22 with collision energies of 13 and 13.3 eV, respectively. The 3xphospho peptide (+2 charges) has a precursor *m*/*z* of 888 and product *m*/*z* of 778.637, 839.28, and 879.113 with collision energies of 21.89, 15.74, and 15.87 eV, respectively. The 1xphospho and 2xphospho peptides (+2 charges) have a precursor *m*/*z* of 808 and 848, with the product *m*/*z* setting of 707.72, 759.22, 778.637, 839.28, and 879.113 (collision energies of 13, 13.3, 21.89, 15.74, and 15.87 eV, respectively).

### Salt fractionation of HCT116 nuclei

Two 15-cm dishes of HCT116 cells were harvested and gently resuspended in 2 ml of buffer 1.A [0.32 M sucrose, 60 mM KCl, 15 mM NaCl, 5 mM MgCl_2_, 0.1 mM EGTA, 15 mM tris (pH 7.4), 0.5 mM DTT, 0.1 mM phenylmethylsulfonyl fluoride (PMSF), and 1× protease inhibitor cocktail]. After mixing with 2 ml of buffer 1.B [0.32 M sucrose, 60 mM KCl, 15 mM NaCl, 5 mM MgCl_2_, 0.1 mM EGTA, 15 mM tris (pH 7.4), and 0.1% NP-40], cells were incubated at 4°C for 10 min to disrupt the plasma membrane. The slurry was added slowly to the surface of 10 ml of precooled buffer 2 [1.2 M sucrose, 60 mM KCl, 15 mM NaCl, 5 mM MgCl_2_, 0.1 mM EGTA, 15 mM tris (pH 7.4), 0.5 mM DTT, 0.1 mM PMSF, and 1× protease inhibitor cocktail] and was centrifuged for 20 min at 10,000*g* at 4°C. The nuclei were resuspended with 1 ml of buffer 3A [10 mM tris (pH 7.4), 2 mM MgCl_2_, 5 mM CaCl_2_, and 0.1 mM PMSF] and digested with 50 U of micrococcal nuclease (MNase) (Thermo Fisher Scientific) at 37°C for 2 hours with gentle rotation. After centrifugation at 500*g* for 1 min, the supernatant was collected as the “0 mM fraction.” The pellet was washed once with 1 ml of buffer 3B [10 mM tris (pH 7.4), 2 mM MgCl_2_, and 0.1 mM PMSF]. The nuclei were further resuspended with 200 μl of buffer 4 [10 mM tris (pH 7.4), 2 mM MgCl_2_, 2 mM EGTA, 0.1% Triton X-100, and 0.1 mM PMSF] with increasing concentrations of NaCl and rotated for 10 min at 4°C before centrifugation at 500*g* for 1 min. The supernatant after salt exaction was collected separately for immunoblotting analysis.

### SDS–polyacrylamide gel electrophoresis and immunoblotting

Cells were washed once with cold PBS and lysed with Laemmli lysis buffer [60 mM tris-HCl (pH 6.8), 10% (v/v) glycerol, and 2% (w/v) SDS] for 5 min at 95°C, and protein concentrations were determined using the BCA assay. Proteins were separated using premade SDS–polyacrylamide gel electrophoresis gels and transferred to polyvinylidene difluoride membranes. Membranes were blocked at room temperature in TBS supplemented with 0.1% (v/v) Tween 20 and 5% milk powder before incubation with primary antibodies at 4°C overnight. Membranes were washed four times in Tris buffered saline with Tween 20 (TBST) and incubated with HRP-conjugated secondary antibodies for 1 hour at room temperature. Membranes were washed three times in TBST and incubated with ECL reagents before image acquisition.

### Immunoprecipitation–mass spectrometry

Five 15-cm dishes of HEK293T or HCT116 cells were harvested for each immunoprecipitation and were incubated with Dignam buffer A [10 mM tris-HCl (pH 7.6), 1.5 mM MgCl_2_, and 10 mM KCl] for 15 min before centrifugation at 4°C (600*g*, 3 min). The cells were lysed with radioimmunoprecipitation assay (RIPA) buffer [25 mM tris-HCl (pH 7.4), 1% NP-40, 0.25% sodium deoxycholate, 150 mM NaCl, and 5% glycerol] and benzonase (25 U/ml) for 30 min at 4°C. The supernatant was collected after centrifugation at 21,000*g* for 20 min and incubated with anti-DYKDDDDK affinity beads (Smart Lifesciences, catalog no. SA042005) for 3 hours at 4°C. Beads were washed with RIPA buffer five times and eluted with 100 mM glycine (pH 2.0). Eluted proteins were reduced and alkylated by 20 mM DTT and 40 mM iodoacetamide at 37°C for 30 min, followed by overnight trypsin digestion at 37°C. Seven microliters of 10% TFA was added to stop the digestion reaction. The acidified peptides were desalted with C18 StageTips and analyzed using an Orbitrap Exploris 480 mass spectrometer equipped with the FAIMS Pro interface. Raw files were processed with Proteome Discoverer 2.4 using a four-stage program. For protein assembling, all proteins with a *q* value higher than 0.01 will receive high confidence, while proteins with a *q* value higher than 0.05 will receive medium confidence. The processed proteomics data were provided for label-free quantification-based differential enrichment analysis using DEP 1.18.0.

### Chromatin immunoprecipitation sequencing with reference exogenous genome

ChIP-Rx experiments were performed as previously described (*58*, *59*). Briefly, 1 × 10^7^ human cells were spiked-in with 1 × 10^6^ to 2 × 10^6^ MEF cells, were fixed with 1% paraformaldehyde in PBS for 10 min, and quenched with 0.125 M glycine for 5 min. Fixed cells were used for nuclear isolation and followed by shearing with the Diagenode Bruptor Plus with the high-power mode for 25 cycles (sonication cycle: 30-s on, 30-s off). The chromatin was immunoprecipitated with 5 μg of individual antibodies and 15 μl of preblocked protein A/G beads (Santa Cruz Biotechnology, catalog no. sc-2003). After washing for three times with wash buffer [50 mM Hepes-KOH (pH 7.5), 300 mM LiCl, 1 mM EDTA, 1.0% NP-40, and 0.7% Na-deoxycholate], the captured DNA was eluted and reverse cross-linked with 200 μl of elution buffer [50 mM tris-HCl (pH 8.0), 10 mM EDTA, 1.0% SDS, and proteinase K (200 μg/ml)] by incubating at 55°C overnight. The DNA was purified by phenol-chloroform extraction and ethanol precipitation.

Library preparation was done using the NEBNext Ultra II DNA library prep kit for Illumina (NEB, catalog no. E7645S), and the libraries were sequenced on a NovaSeq 6000. Reads were aligned to the human reference genome GRCh38/hg38 and mouse reference genome GRCm38/mm10 with Bowtie2, allowing only uniquely mapping reads with up to two mismatches. The aligned human reads were normalized using the aligned mouse reads. The human BAM files were normalized and converted to bigwig files using Samtools. ChIP peaks were called using MACS2 (model-based analysis of ChIP-seq) version 2.1.2 with default parameters. Heatmaps and metaplots were made for the indicated windows using the average coverage (reads per million) using Deeptools 2.0. Ngsplot was used to calculate the ratio of PAF1C occupancy to Pol II coverage.

### Quick precision run-on sequencing

PRO-seq was performed as previously reported (*58*, *60*). A total of 1 × 10^6^ cells were used for each run-on reaction, and PRO-seq libraries were sequenced on a NovaSeq 6000 platform. PRO-seq reads were aligned to the human hg38 genome using Bowtie2. The resulting reads were normalized to the total mapped reads (r.p.m.) and converted to bigwig files for visualization in the UCSC Genome Browser. Heatmaps and metaplots were made for the indicated windows using the strand-specific average coverage using Deeptools 2.0.

### Transient transcriptome sequencing

TT-seq was performed as previously described (*50*) with some modifications. Cells were labeled in medium with 400 μM 4-thiouridine (4sU) (Sigma-Aldrich, catalog no. T4509) for 10 min before harvest, and total RNA was extracted with TRIzol (Invitrogen, catalog no. 15596-018). The labeled RNA (100 μg) was fragmented with NaOH, and a spike-in of 10% S2 RNA (10 μg) was used. After biotinylated in a 300-μl reaction [100 μg of RNA, 10 mM Hepes-KOH (pH 7.5), 1 mM EDTA, and MTSEA-biotin-XX (0.167 mg/ml; Sigma-Aldrich, catalog no. M9938)], the biotin-labeled RNA with streptavidin M280 beads (Invitrogen, Cat# 11206D) was enriched and eluted by 100 mM DTT. RNA was purified by 1.8× RNAClean XP beads before library preparation.

Library preparation was done using the NEBNext Ultra II Directional RNA Library Prep Kit (NEB, catalog no. E7760L), and the libraries were sequenced on a NovaSeq 6000. TT-seq reads were aligned to the human reference genome GRCh38/hg38 or *Drosophila* reference genome BDGP Release 6/dm6 with Bowtie2. The aligned human reads were normalized using the aligned *Drosophila* reads. The human BAM files were normalized and converted to bigwig files using Deeptools 2.0. Heatmaps, metaplots, and boxplots were made for the indicated windows using the average coverage.

### HaloPROTAC3 synthesis

#### Synthesis of 2-hydroxy-4-(4-methylthiazol-5-yl)benzonitrile compound 2

Compound 1 (5 g, 25 mmol), potassium acetate (4.9 g, 50 mmol), and Pd(OAc)_2_ (1.7 mg, 0.008 mmol) were added to 15 ml of Dimethylacetamide (DMAC) in a 50-ml round-bottom flask. The flask was degassed and refilled with argon three times. 4-Methylthiazole (4.6 ml, 50 mmol) was added, and the resulting mixture was allowed to stir at 150°C overnight; the mixture was cooled to room temperature and diluted with water. The aqueous layer was extracted three times with CH_2_Cl_2_. The combined extracts were washed with brine, dried over Na_2_SO_4_, filtered, and concentrated. Purification by column chromatography gave a yellow solid (4.13 g, 76%).^**1**^**H NMR** (400 MHz, acetone) δ 8.97 (s, 1H), 7.70 (d, *J* = 8.1 Hz, 1H), 7.23 (d, *J* = 1.6 Hz, 1H), 7.16 (dd, *J* = 8.1, 1.7 Hz, 1H), 2.53 (s, 3H).

#### Synthesis of 2-(aminomethyl)-5-(4-methylthiazol-5-yl)phenol compound 3

Lithium aluminum hydride (1.68 g, 44.4 mmol) was added to a 100-ml round-bottom flask. The flask was placed in an ice bath, and anhydrous tetrahydrofuran (THF) (15 ml) was added slowly and then degassed and refilled with argon three times. Compound **2** (1.6 g, 7.4 mmol) was dissolved in a separate THF (10 ml) flask, transferred slowly to the solution of lithium aluminium hydride (LAH), and washed once with THF (5 ml). The mixture was then heated to 50°C overnight. Thin-layer chromatography (TLC) showed that compound **2** was consumed completely. The mixture was cooled with ice water and quenched with water (2 × 3 ml) followed by 3 M sodium hydroxide (2 × 3 ml). The mixture was filtered through filter paper and washed three times with THF. The organic layer was concentrated and purified by column chromatography to give a yellow oil (0.483 g, 30%).^**1**^**H NMR** (400 MHz, methanol) δ 8.85 (s, 1H), 7.24 (d, *J* = 7.6 Hz, 1H), 6.91 (d, *J* = 1.8 Hz, 1H), 6.85 (dd, *J* = 7.7, 1.6 Hz, 1H), 3.96 (s, 2H), 2.49 (s, 3H).

#### Synthesis of tert-butyl (2S,4R)-4-hydroxy-2-{[2-hydroxy-4-(4-methylthiazol-5-yl)benzyl]carbamoyl}pyrrolidine-1-carboxylate compound 4

Boc-l-hydroxyproline (0.642 g, 2.78 mmol) was added to 15 ml of anhydrous *N*,*N*′-dimethylformamide (DMF) in a 50-ml round-bottom flask. The flask was degassed and refilled with argon three times. *N*,*N*-Diisopropylethylamine (1.5 ml, 2.78 mmol) was added dropwise, then followed by hexafluorophosphate azabenzotriazole tetramethyl uronium (HATU) (1.16 g, 3.06 mmol), and reacted at room temperature for 30 min. Then compound **3** (0.631 g, 2.78 mmol) was added, and the resulting mixture was stirred for another 3 hours. After the reaction, the compounds were extracted with ethyl acetate (3 × 30 ml) and washed with brine, dried over Na_2_SO_4_, filtered, and concentrated. Purification by column chromatography gave a yellow solid (0.756 g, 62%). ^**1**^**H NMR** (400 MHz, CDCl_3_) δ 8.65 (s, 1H), 7.17 (d, *J* = 7.8 Hz, 1H), 7.01 to 6.93 (m, 1H), 6.89 to 6.82 (m, 1H), 4.44 to 4.28 (m, 4H), 3.59 to 3.49 (m, 2H), 3.27 (d, *J* = 2.9 Hz, 1H), 2.50 to 2.45 (m, 3H), 2.31 (s, 1H), 1.29 to 1.21 (m, 3H), 1.14 (s, 6H).

#### Synthesis of (2S,4R)-4-hydroxy-N-[2-hydroxy-4-(4-methylthiazol-5-yl)benzyl]pyrrolidine-2-carboxamide compound 5

TFA (3 ml) was added to a solution of compound 4 (0.756 mg, 1.7 mmol) in dichloromethane (DCM) (6 ml) at 0°C. The reaction mixture was slowly warmed to room temperature. TLC analysis indicated that the reaction was completed within 1 hour. The reaction mixture was concentrated in vacuo, and the crude product **5** was used for the next step without further purification.

#### Synthesis of (S)-3-methyl-2-(1-oxoisoindolin-2-yl)butanoic acid compound 8

l-valine (3.42 g, 29.2 mmol) and phthalaldehyde (4.3 g, 32 mmol) were added to a 30-ml anhydrous CH_3_CN in a 100-ml round-bottom flask. The flask was fitted with a reflux condenser and heated in a 90°C oil bath for 3.5 hours. After the reaction was complete, the mixture was then cooled to room temperature and then to 4°C. The mixture was filtered and washed with cold CH_3_CN. The resulting light yellow crystals were then dried under vacuum to give the desired product (5.40 g, 80%). ^**1**^**H NMR** (400 MHz, DMSO-*d*_6_) δ 7.72 (d, *J* = 7.5 Hz, 1H), 7.63 (d, *J* = 4.1 Hz, 2H), 7.51 (dq, *J* = 8.0, 4.1 Hz, 1H), 4.64 (d, *J* = 17.7 Hz, 1H), 4.57 to 4.49 (m, 2H), 1.02 (d, *J* = 6.6 Hz, 3H), 0.85 (d, *J* = 6.7 Hz, 3H).

#### Synthesis of (2S,4R)-4-hydroxy-N-[2-hydroxy-4-(4-methylthiazol-5-yl)benzyl]-1-[(S)-3-methyl-2-(1-oxoisoindolin-2-yl)butanoyl]pyrrolidine-2-carboxamide compound 9

Compound 8 was added (0.089 g, 0.38 mmol) to 5-ml anhydrous DMF in a 50-ml round-bottom flask. The flask was degassed and refilled with argon three times. *N*,*N*-Diisopropylethylamine (0.22 ml, 1.2 mmol) was added dropwise, then added with HATU (0.138 g, 0.363 mmol), and reacted at room temperature for 30 min. Then, compound 5 (0.128 g, 0.346 mmol) was added, and the resulting mixture was stirred for another 3 hours. After the reaction was complete, the products were extracted with ethyl acetate (3 × 30 ml). The combined extracts were washed with brine, dried over Na_2_SO_4_, filtered, and concentrated. Purification by column chromatography was done to give a cream-colored solid (0.0592 mg, 31%). ^**1**^**H NMR** (400 MHz, methanol) δ 8.85 (s, 1H), 7.77 (d, *J* = 7.4 Hz, 1H), 7.66 to 7.55 (m, 2H), 7.52 (dd, *J* = 8.1, 2.9 Hz, 1H), 7.50–7.45 (m, 1H), 7.36 (d, *J* = 7.7 Hz, 1H), 6.96 to 6.87 (m, 2H), 4.87 (d, *J* = 11.0 Hz, 1H), 4.68 to 4.41 (m, 7H), 4.02 (d, *J* = 11.1 Hz, 1H), 3.91 (dd, *J* = 11.0, 4.0 Hz, 1H), 2.48 (s, 3H), 2.47 to 2.40 (m, 1H), 2.22 (ddt, *J* = 11.9, 7.9, 2.0 Hz, 1H), 2.10 (td, *J* = 8.9, 4.4 Hz, 1H), 1.08 (t, *J* = 6.0 Hz, 3H), 0.82 (d, *J* = 6.7 Hz, 3H).

#### Synthesis of 2-(2-{2-[(6-chlorohexyl)oxy]ethoxy}ethoxy)ethan-1-ol compound 12

NaH (0.2 g, 5 mmol, 60% dispersion in mineral oil) was added to a 50-ml round-bottom flask with anhydrous DMF (5 ml) and THF (5 ml). The flask was degassed and refilled with argon three times. Compound **10** (1.5 g, 10 mmol) was added dropwise to the mixture under an ice bath. After 40 min, compound **11** (493 mg, 2 mmol) was added and the mixture was warmed to room temperature and stirred overnight. After TLC detected, the reaction was complete; the mixture was quenched with water, diluted with 1 M HCl, and extracted with chloroform (3 × 30 ml). The combined extracts were washed with brine, dried over Na_2_SO_4_, filtered, and concentrated. Purification by column chromatography was done to give the monoalkylated product (0.270 mg, 50%).^**1**^**H NMR** (400 MHz, CDCl_3_) δ 3.69 (s, 2H), 3.64 (d, *J* = 7.2 Hz, 6H), 3.57 (dt, *J* = 9.8, 4.4 Hz, 4H), 3.51 (t, *J* = 6.7 Hz, 2H), 3.44 (t, *J* = 6.7 Hz, 2H), 1.76 (q, *J* = 7.0 Hz, 2H), 1.62 to 1.54 (m, 2H), 1.47 to 1.31 (m, 4H).

#### Synthesis of 2-(2-{2-[(6-chlorohexyl)oxy]ethoxy}ethoxy)ethyl 4-methylbenzenesulfonate compound 13

Compound 12 (0.168 g, 0.62 mmol) was added to a 5-ml CH_2_Cl_2_ in a 50-ml round-bottom flask at room temperature. Triethylamine (0.189 g, 1.86 mmol) and tosyl chloride (180 mg, 0.94 mmol) were added, and the solution was stirred overnight. The mixture was then diluted with 10% citric acid and extracted with CH_2_Cl_2_ (3 × 10 ml). The combined extracts were washed with brine, dried over Na_2_SO_4_, filtered, and concentrated. Purification by column chromatography was done to give compound 13. ^**1**^**H NMR** (400 MHz, chloroform-*d*) δ 7.81 to 7.71 (m, 2H), 7.31 (d, *J* = 8.0 Hz, 2H), 4.12 (dd, *J* = 5.6, 4.0 Hz, 2H), 3.66 to 3.63 (m, 2H), 3.58 to 3.52 (m, 8H), 3.49 (t, *J* = 6.7 Hz, 2H), 3.41 (t, *J* = 6.6 Hz, 2H), 2.41 (s, 3H), 1.72 (dt, *J* = 8.5, 6.7 Hz, 2H), 1.55 (d, *J* = 14.4 Hz, 2H), 1.44 to 1.38 (m, 2H), 1.34 (tdd, *J* = 6.8, 4.8, 1.7 Hz, 2H).

#### Synthesis of (2S,4R)-N-{2-[2-(2-{2-[(6-chlorohexyl)oxy]ethoxy}ethoxy)ethoxy]-4-(4-methylthiazol-5-yl)benzyl}-4-hydroxy-1-[(S)-3-methyl-2-(1-oxoisoindolin-2-yl)butanoyl]pyrrolidine-2-carboxamide 14

Compound **9** (0.048 mg, 0.086 mmol) and compound **13** (0.044 mg, 0.1 mmol) were added to a 5-ml DMF in a 50-ml round-bottom flask. Potassium carbonate (0.030 mg, 0.21 mmol) was then added, and the mixture was heated to 70°C and stirred overnight. The mixture was cooled to room temperature and diluted with water. The aqueous layer was extracted with EtOAc (3 × 10 ml). The combined extracts were washed with brine, dried over Na_2_SO_4_, filtered, and concentrated. Purification by column chromatography was done to give compound **14**. ^**1**^**H NMR** (400 MHz, chloroform-*d*) δ 8.61 (s, 1H), 7.58 (d, *J* = 7.6 Hz, 1H), 7.50 (t, *J* = 6.0 Hz, 1H), 7.33 (d, *J* = 7.5 Hz, 1H), 7.28 (d, *J* = 5.9 Hz, 1H), 7.21 (s, 2H), 6.88 (d, *J* = 7.7 Hz, 1H), 6.80 (s, 1H), 4.72 (q, *J* = 14.3, 12.6 Hz, 2H), 4.56 (d, *J* = 9.6 Hz, 1H), 4.49 to 4.39 (m, 2H), 4.35 to 4.23 (m, 2H), 4.18 to 4.09 (m, 3H), 3.84 (dq, *J* = 15.9, 6.0 Hz, 2H), 3.71 (dd, *J* = 11.1, 3.9 Hz, 1H), 3.66 (dd, *J* = 5.8, 3.5 Hz, 2H), 3.58 (dd, *J* = 5.9, 3.5 Hz, 2H), 3.52 (dd, *J* = 5.8, 3.6 Hz, 2H), 3.46 to 3.40 (m, 4H), 3.32 (t, *J* = 6.6 Hz, 2H), 2.42 (s, 3H), 2.34 to 2.26 (m, 1H), 2.15 (ddd, *J* = 12.8, 8.3, 4.7 Hz, 1H), 2.02 (dd, *J* = 13.1, 8.1 Hz, 1H), 1.66 (q, *J* = 7.0 Hz, 2H), 1.50 to 1.44 (m, 2H), 1.33 (dd, *J* = 10.8, 5.1 Hz, 2H), 1.25 (q, *J* = 7.7 Hz, 2H), 0.91 (d, *J* = 6.5 Hz, 3H), 0.72 (d, *J* = 6.6 Hz, 3H).

### Statistical analysis

Data are presented as means ± SD. All quantitative results were analyzed with the test indicated in the figure legends, after confirming that the data met appropriate assumptions (normality, homogeneous variance, and independent sampling). The peak or gene size (*N*) in the heatmaps indicates the number of regions or genes included. The sample size (*n*) indicates the number of technical replicates. For fig. S2D, one-way analysis of variance (ANOVA) tests were performed with Prism 7 (GraphPad Software, La Jolla, CA) to determine the statistical significance. For Figs. 3G and 5D, a two-tailed unpaired *t* test was used for statistical analysis. For Figs. 4H and 6G, the statistical significance was determined by the Wilcoxon signed-rank test using R 3.2.1 package. For Fig. 6 (D and E), IC_50_ values were calculated by nonlinear fitting with four parameters with Prism 7.

## Notes

View/request a protocol for this paper from *Bio-protocol*.
