# Supplementary material for: CDK12 and Integrator-PP2A complex modulates LEO1 phosphorylation for processive transcription elongation
Source: Sci Adv. 2023 May 19;9(20):eadf8698. doi: 10.1126/sciadv.adf8698 (PMC10198629; doi:10.1126/sciadv.adf8698)
Supplement: Supplementary file 1 — Figs. S1 to S5 Legends for tables S1 to S4 [file sciadv.adf8698_sm.pdf]

Science Advances
AAAS

Supplementary Materials for

CDK12 and Integrator-PP2A complex modulates LEO1 phosphorylation for processive transcription elongation

Min Qiu *et al.*

Corresponding author: Kaiwei Liang, kwliang@whu.edu.cn; Ruijing Xiao, xrj7619@whu.edu.cn; Pingping Fang, pingpingfang@whu.edu.cn

Sci. Adv. **9**, eadf8698 (2023)
DOI: 10.1126/sciadv.adf8698

The PDF file includes:

Figs. S1 to S5
Legends for tables S1 to S4

Other Supplementary Material for this manuscript includes the following:

Tables S1 to S4

A

Phosphorylated proteins identified by *in situ* phosphorylation assays

Protein	Phosphorylated sites
SMARCA4	S1627 S1631 S1570 S1575
HDGFL2	S305 S307 S370 S192 S303
LEO1	S66 S151 S154 S279 S608
TCOF1	S764 S765 S341 S342 S343 S272 S273
NOLC1	S366 S368 S84 S90 S91 S470 S326
CTNNA1	S652 T654 S655
SRRM2	S1382 S1383 S1387
NCL	S34 S28 S41 S42 S145
SUPT5H	T799 T806
SRRM1	T872 S616
DDX54	S39 S41
HMGA1	S102 S103
POLR2A	S1913 S1927
RAB11FIP1	S202 S206
FTSJ3	S333 S335
NOM1	S280 T287
GTF2F1	S218 S351
CANX	S554 S564
PRPF40A	S888 T932
TJP1	S337 S617 S125
ZRANB2	S120 S153
CPD	T1368

Protein	Sites
CDC5L	T396
NDUFA4	S66
HTATSF1	S600
RSF1	S1282
SSFA2	S270
SON	S1782
RBM5	S78
MISP	S394
TRA2A	S2
MTDH	S298
OCIAD1	S209
KRI1	S171
PRPF4B	S142
UTP18	S124
ANKRD11	S577
PPP1CC	T311
THRAP3	S939
PRPF38A	S193
PPIG	T748
RBM25	S677
LMNA	S22
TFAM	S124

Protein	Sites
RRP12	S1080
BCLAF1	S397
RTF1	S313
HDAC2	S394
DHX16	S103
CTR9	S1130
HIRIP3	S363
SURF2	T195
MYBBP1A	S738
BMS1	S494
PGAM5	S87
BRD2	T629
EIF2S2	S67
ACIN1	S216
RBM17	S155
PPP1R12C	T560
PPP1R12A	T696
TMX1	S247
MCM2	S139
AP1M1	T154
CDK11A	S271

B

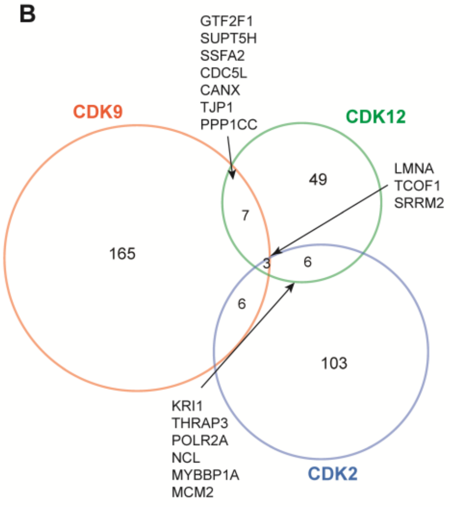

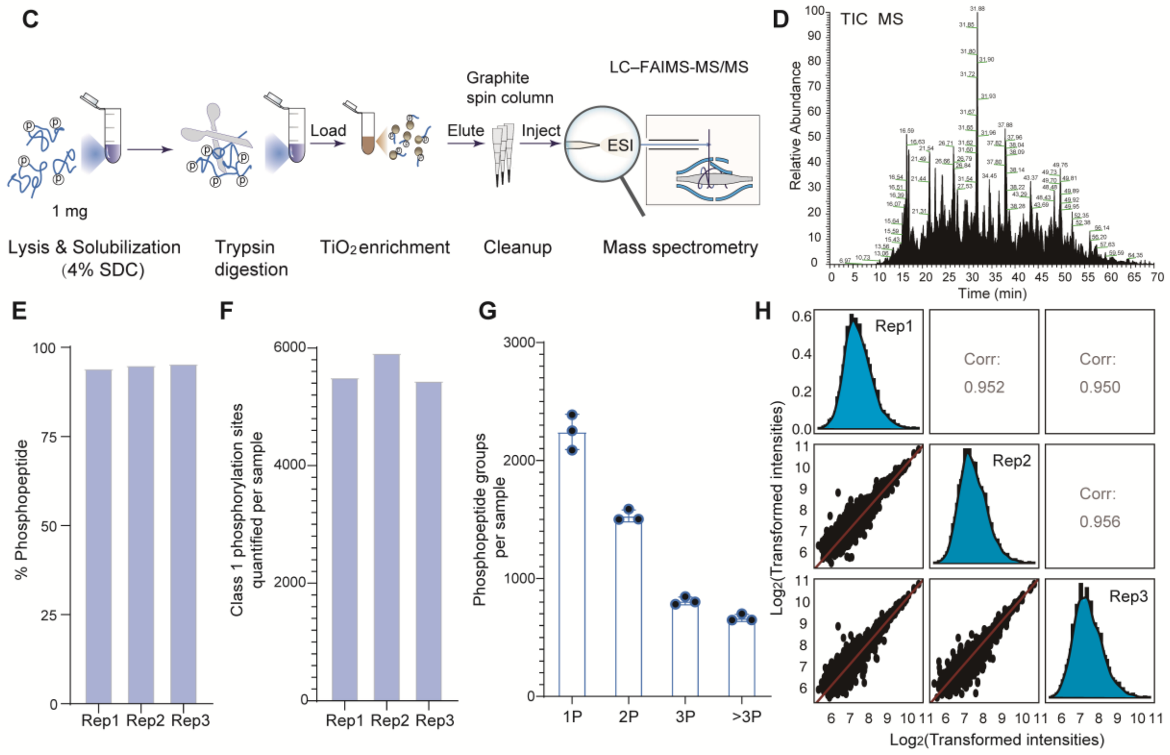

Figure S1

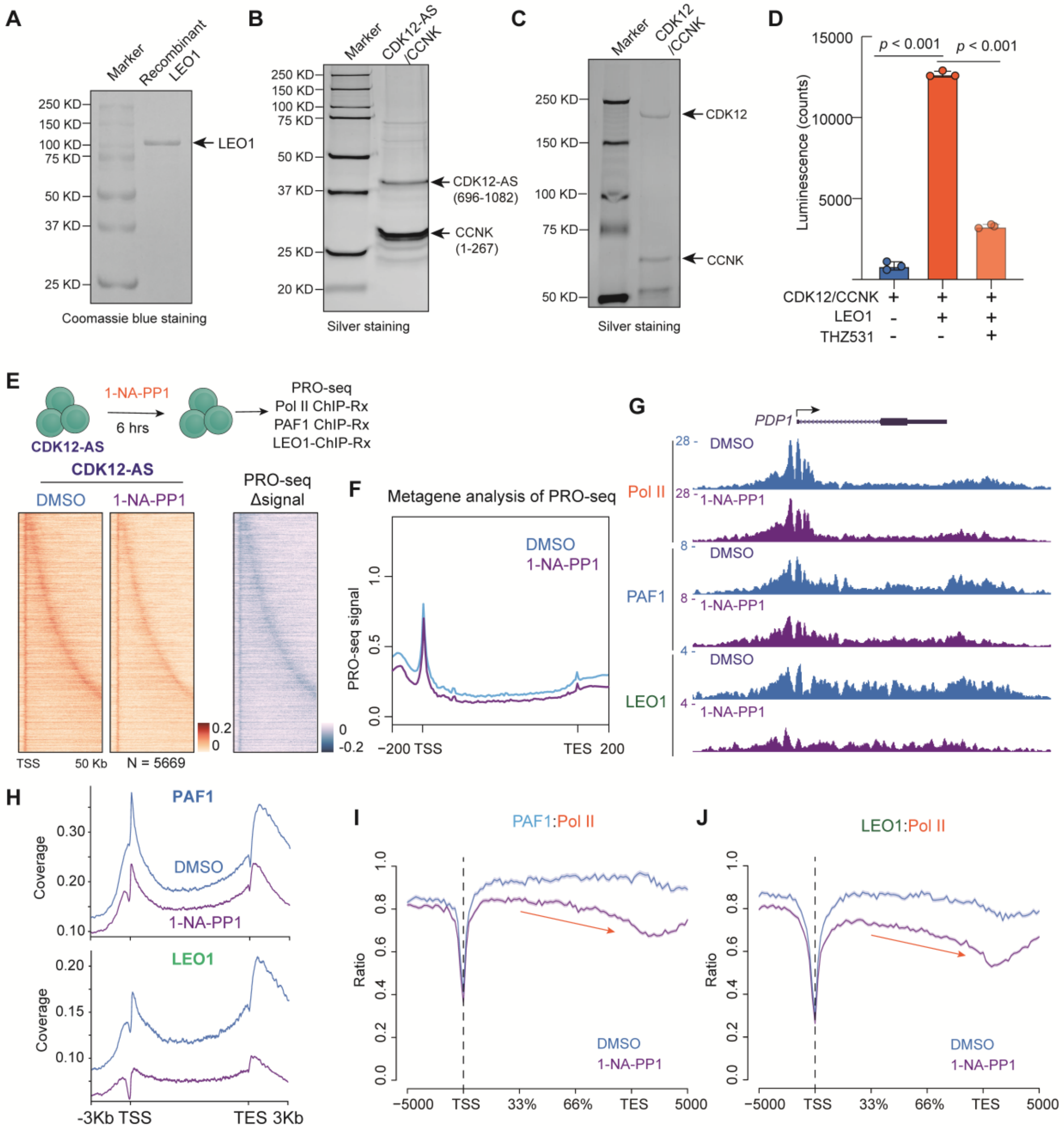

A
Marker
Recombinant LEO1
250 KD
150 KD
100 KD
75 KD
50 KD
37 KD
25 KD
LEO1
Coomassie blue staining
B
Marker
CDK12-AS /CCNK
250 KD
150 KD
100 KD
75 KD
50 KD
37 KD
25 KD
20 KD
CDK12-AS (696-1082)
CCNK (1-267)
Silver staining
C
Marker
CDK12 /CCNK
250 KD
150 KD
100 KD
75 KD
50 KD
CDK12
CCNK
Silver staining
D
p < 0.001
p < 0.001
Luminescence (counts)
15000
10000
5000
CDK12/CCNK + + +
LEO1 - + +
THZ531 - - +
E
1-NA-PP1
6 hrs
CDK12-AS
PRO-seq
Pol II ChIP-Rx
PAF1 ChIP-Rx
LEO1-ChIP-Rx
CDK12-AS
DMSO
1-NA-PP1
PRO-seq Δsignal
0.2
0
-0.2
TSS
50 Kb
N = 5669
F
Metagene analysis of PRO-seq
DMSO
1-NA-PP1
PRO-seq signal
−200 TSS
TES 200
G
PDP1
Pol II
PAF1
LEO1
DMSO
1-NA-PP1
28
8
4
H
PAF1
LEO1
Coverage
DMSO
1-NA-PP1
-3Kb TSS
TES 3Kb
I
PAF1:Pol II
Ratio
DMSO
1-NA-PP1
−5000 TSS 33% 66% TES 5000
J
LEO1:Pol II
Ratio
DMSO
1-NA-PP1
−5000 TSS 33% 66% TES 5000

Figure S2

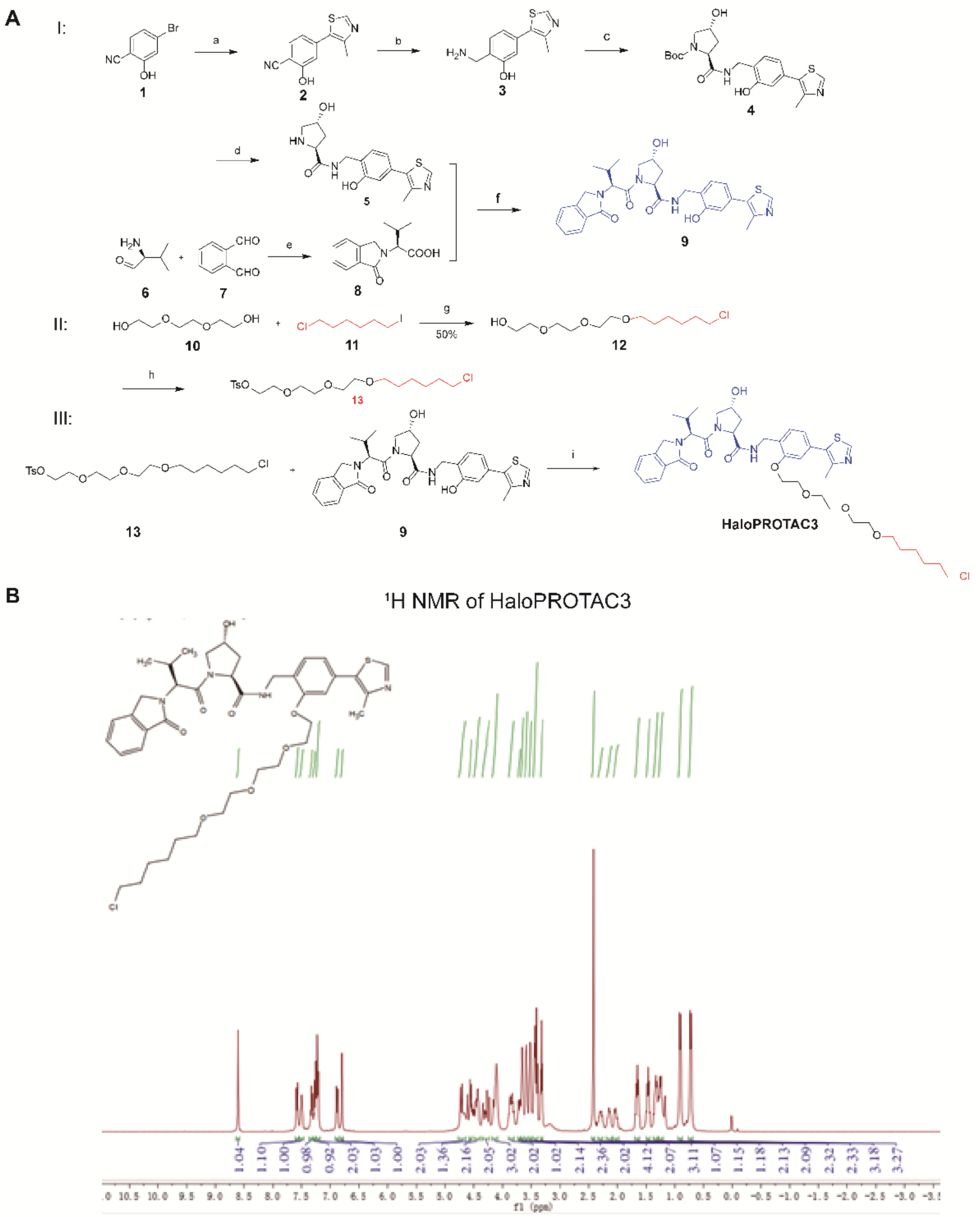

Figure S3

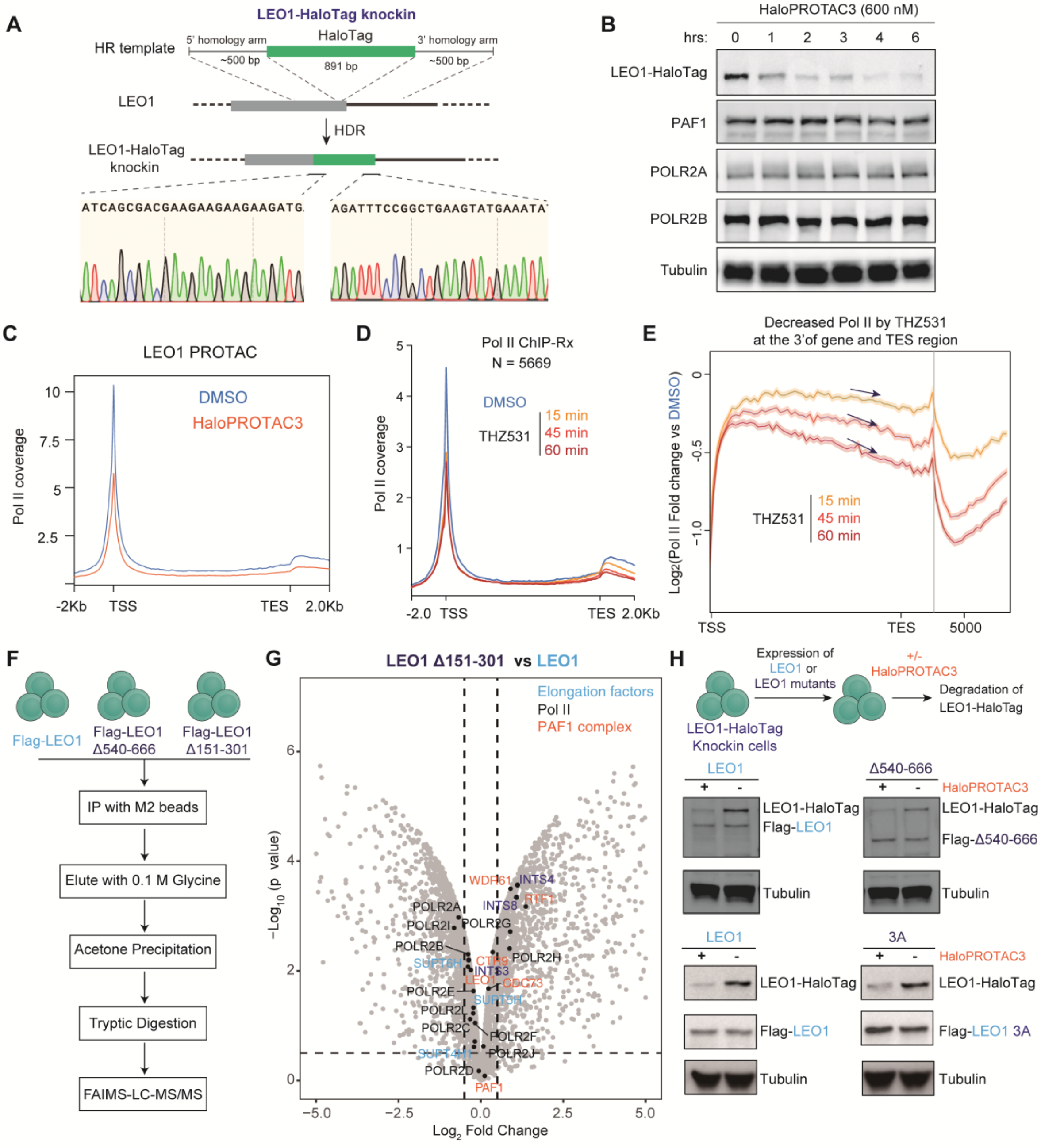

A
LEO1-HaloTag knockin
HR template
5' homology arm
HaloTag
3' homology arm
~500 bp
891 bp
~500 bp
LEO1
HDR
LEO1-HaloTag knockin
ATCAGCGACGAAGAAGAAGAAGATG
AGATTTCCGGCTGAAGTATGAAATA
B
HaloPROTAC3 (600 nM)
hrs: 0 1 2 3 4 6
LEO1-HaloTag
PAF1
POLR2A
POLR2B
Tubulin
C
LEO1 PROTAC
DMSO
HaloPROTAC3
Pol II coverage
-2Kb TSS TES 2.0Kb
D
Pol II ChIP-Rx
N = 5669
DMSO
THZ531
15 min
45 min
60 min
Pol II coverage
-2.0 TSS TES 2.0Kb
E
Decreased Pol II by THZ531 at the 3'of gene and TES region
Log2(Pol II Fold change vs DMSO)
THZ531
15 min
45 min
60 min
TSS TES 5000
F
Flag-LEO1
Flag-LEO1 Δ540-666
Flag-LEO1 Δ151-301
IP with M2 beads
Elute with 0.1 M Glycine
Acetone Precipitation
Tryptic Digestion
FAIMS-LC-MS/MS
G
LEO1 Δ151-301 vs LEO1
Elongation factors
Pol II
PAF1 complex
−Log10 (p value)
Log2 Fold Change
H
Expression of LEO1 or LEO1 mutants
+/- HaloPROTAC3
Degradation of LEO1-HaloTag
LEO1-HaloTag Knockin cells
LEO1
Δ540-666
HaloPROTAC3
LEO1-HaloTag
Flag-LEO1
Flag-Δ540-666
Tubulin
3A
Flag-LEO1 3A

Figure S4

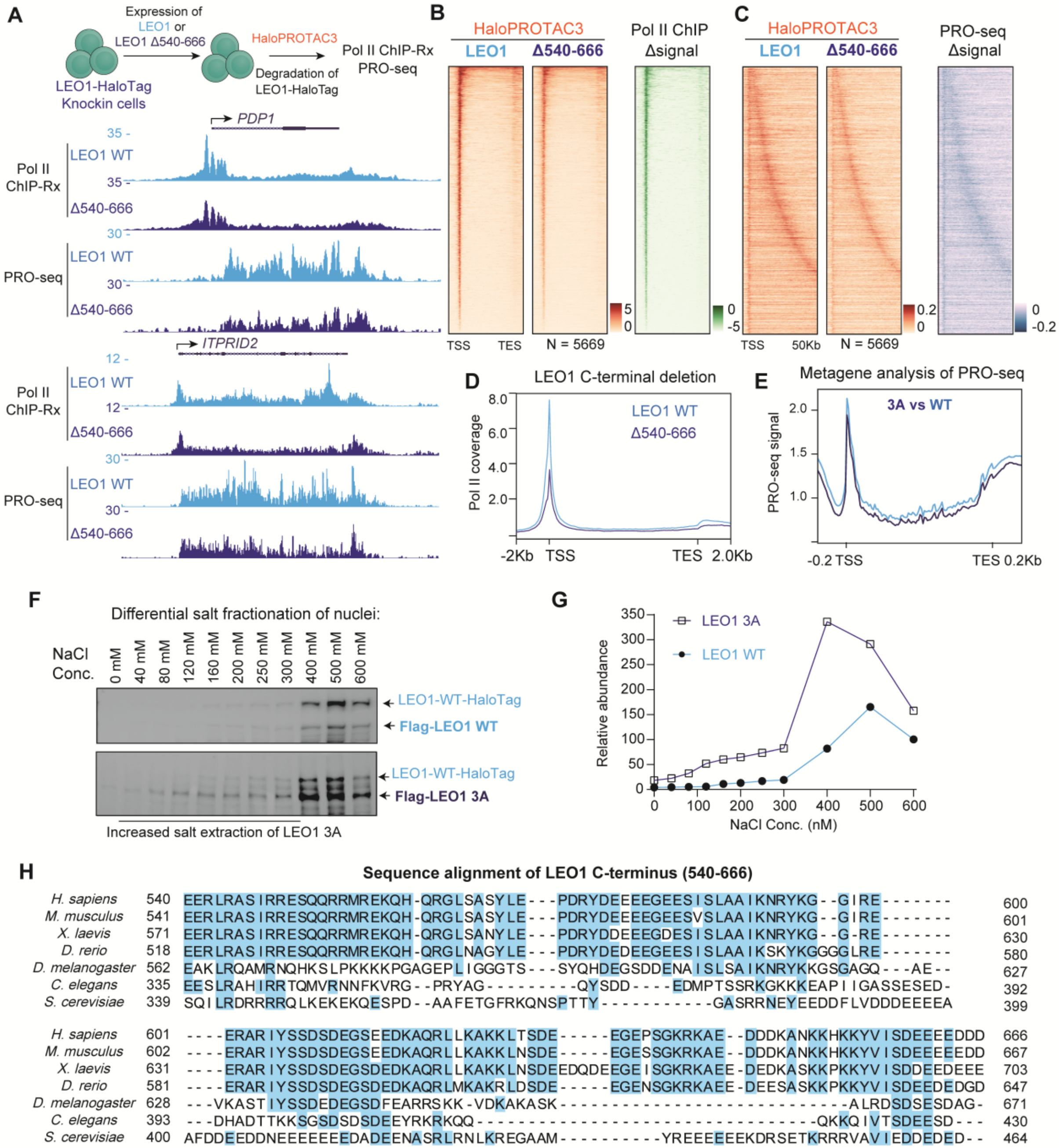

A
Expression of LEO1 or LEO1 Δ540-666
HaloPROTAC3
Degradation of LEO1-HaloTag
Pol II ChIP-Rx
PRO-seq
LEO1-HaloTag Knockin cells
PDP1
ITPRID2
Pol II ChIP-Rx
PRO-seq
LEO1 WT
Δ540-666
B
HaloPROTAC3
LEO1
Δ540-666
Pol II ChIP Δsignal
TSS
TES
N = 5669
C
PRO-seq Δsignal
50Kb
D
LEO1 C-terminal deletion
Pol II coverage
-2Kb
2.0Kb
E
Metagene analysis of PRO-seq
3A vs WT
PRO-seq signal
-0.2 TSS
TES 0.2Kb
F
Differential salt fractionation of nuclei:
NaCl Conc.
0 mM
40 mM
80 mM
120 mM
160 mM
200 mM
250 mM
300 mM
400 mM
500 mM
600 mM
LEO1-WT-HaloTag
Flag-LEO1 WT
Flag-LEO1 3A
Increased salt extraction of LEO1 3A
G
LEO1 3A
LEO1 WT
Relative abundance
NaCl Conc. (nM)
H
Sequence alignment of LEO1 C-terminus (540-666)
H. sapiens 540 EERLRASIRRESQQRRMREKQH-QRGLSASYLE---PDRYDEEEEGEESISLAAIKNRYKG--GIRE------- 600
M. musculus 541 EERLRASIRRESQQRRMREKQH-QRGLSASYLE---PDRYDEEEEGEESVSLAAIKNRYKG--GIRE------- 601
X. laevis 571 EERLRASIRRESQQRRMREKQH-QRGLSANYLE---PDRYDDEEEGDESISLAAIKNRYKG--G-RE------- 630
D. rerio 518 EERLRASIRRESQQRRMREKQH-QRGLNAGYLE---PDRYDEDEEGEESISLAAIKSKYKGGGGLRE------- 580
D. melanogaster 562 EAKLRQAMRNQHKSLPKKKKPGAGEPLIGGGTS---SYQHDEGSDDENAISLSAIKNRYKKGSGAGQ---AE-- 627
C. elegans 335 EESLRAHIRRTQMVRNNFKVRG--PRYAG---------QYSDD----EDMPTSSRKGKKKEAPIIGASSESED- 392
S. cerevisiae 339 SQILRDRRRRQLKEKEKQESPD--AAFETGFRKQNSPTTY-----------GASRRNEYEEDDFLVDDDEEEEA 399
H. sapiens 601 ----ERARIYSSDSDEGSEEDKAQRLLKAKKLTSDE-----EGEPSGKRKAE--DDDKANKKHKKYVISDEEEEDDD 666
M. musculus 602 ----ERARIYSSDSDEGSEEDKAQRLLKAKKLNSDE-----EGESSGKRKAE--DDDKANKKHKKYVISDEEEEEDD 667
X. laevis 631 ----ERARIYSSDSDEGSDEDKAQRLLKAKKLNSDEEDQDEEGEISGKRKAEE-DEDKASKKPKKYVISDDEEDEEE 703
D. rerio 581 ----ERARIYSSDSDEGSDEDKAQRLMKAKRLDSDE-----EGENSGKRKAEE-DEESASKKPKKYVISDEEDEDGD 647
D. melanogaster 628 ---VKASTIYSSDEDEGSDFEARRSKK-VDKAKASK---------------------------ALRDSDSESDAG- 671
C. elegans 393 ---DHADTTKKSGSDSDSDEEYRKRKQQ-------------------------------QKKQIVTSDEESD--- 430
S. cerevisiae 400 AFDDEEDDNEEEEEEEDADEENASRLRNLKREGAAM----------YREEEEEEKDRSETKRRRVAVIEDDEDED-- 464

Figure S5

SUPPLEMENTARY FIGURES

Fig S1. Identification of CDK12 substrates by *in situ* nuclear phosphorylation and phosphoproteomic analysis.

(A) Phosphorylated proteins identified by *in situ* phosphorylation assays. **(B)** Comparison of identified CDK12, CDK9 (*22*), and CDK2 (*37*) substrates. **(C)** Schematic of phosphoproteomic analysis using titanium dioxide (TiO_2) enrichment and mass spectrometry. Cells were lysed in 4% sodium deoxycholate (SDC) buffer, digested with trypsin, and enriched with TiO_2 beads, followed by graphite spin column cleanup and LC–FAIMS-MS/MS analysis (*47*). **(D)** Total ion chromatogram (TIC) of MS1 intensity during the acquisition process. **(E)** Percentage of phosphopeptides in three replicates. Around 95% of the peptides identified by mass spectrometry were phosphorylated, indicating the effective enrichment of phosphopeptides by TiO_2 beads. **(F)** Number of quantified class 1 phosphorylation sites (localization probability > 0.75) in each replicate. **(G)** Number of phosphopeptide groups containing one (1P), two (2P), three (3P), or more than three phosphate groups. **(H) S**catterplots depicting the log_2-transformed intensities of phosphopeptides from three replicate experiments. Pearson correlation coefficients between replicates are shown.

Fig S2. Characterization of transcription elongation factor LEO1 as a bona fide substrate of CDK12 kinase

(A) Coomassie blue staining of recombinant full-length LEO1 proteins purified from *E.coli*. **(B)** Silver staining of CDK12-AS/CCNK complex purified from HEK293T cells. **(C)** Silver staining of full-length CDK12/CCNK complex purified from HEK293T cells. **(D)** ADP-Glo kinase assays of full-length CDK12/CCNK on LEO1 phosphorylation. Quantitation of ATP to ADP conversion by recombinant CDK12/CCNK incubated with recombinant LEO1 was plotted. The phosphorylation of LEO1 by CDK12 is sensitive to the THZ531 inhibitor. Statistical analysis was performed using the one-way ANOVA test.
(E-F) PRO-seq of CDK12-AS cells after DMSO or 1-NA-PP1 treatments for 6 hours. Heatmaps of PRO-seq signals were depicted at all of the LEO1-occupied genes (N=5,669), showing that CDK12 inhibition reduced elongating Pol II at the 3'-end of genes and gene bodies (F). **(G)** UCSC genome browser snapshots of Pol II, PAF1, and LEO1 ChIP-Rx at *PDP1* gene in CDK12-AS cells after 6-hour 1-NA-PP1 treatment. **(H)** Rescaled metagene analysis of PAF1 and LEO1 ChIP-Rx signals (r.p.m) at LEO1-occupied genes in

CDK12-AS cells after DMSO or 1-NA-PP1 treatment. CDK12 inhibition decreases the genome-wide occupancy of PAF1C across the gene bodies. **(I and J)** Meta-analyses of scaled LEO1-occupied genes with ratios of PAF1 (I) or LEO1 (J) to Pol II after treatment of CDK12-AS cells with DMSO or 1-NA-PP1 for 6 hours. CDK12 inhibition decreases the ratio of PAF1C coverage (r.p.m) to Pol II across the gene body to TES.

Fig S3. Synthesis of HaloPROTAC3.

(A) Scheme for chemical synthesis of HaloPROTAC3. Reagents and conditions: (a) 4-methyl-thiazole, $Pd(OAc)_2$, KOAc, DMAC, 150 °C, overnight; (b) LAH, THF, 50 °C, overnight; (c) Boc-HypOH, DIPEA, HATU, DMF, r.t., 2 h; (d) DCM:TFA = 1:1, r.t., 1h; (e) MeCN, 90 °C, r.t., 3.5 h; (f) DIPEA, HATU, DMF, r.t., 3 h; (g) NaH, THF:DMF = 1:1, r.t., overnight; (h) TsCl, Et_3N, DCM, r.t., overnight; (i) DMF, K_2CO_3, 70 °C, overnight. **(B)** ^{1}H NMR of HaloPROTAC3.

Fig S4. CDK12 phosphorylates LEO1 for processive transcription elongation.

(A) Development of LEO1-HaloTag knockin cells by CRISPR-Cas9 and HDR. HaloTag coding sequence was inserted into the 3'-end of the LEO1 coding gene and was confirmed by Sanger sequencing. **(B)** LEO1-HaloTag knockin cells were treated with 600 nM HaloPROTAC3 (*49*) for various periods before immunoblotting with anti-HaloTag, anti-PAF1, anti-POLR2A, and anti-POLR2B. Tubulin serves as a loading control. **(C)** Metagene analysis of Pol II occupancy (spike-in normalized r.p.m) after acute LEO1 degradation. **(D)** Pol II ChIP-Rx of HCT116 cells after 500 nM THZ531 treatment for 0-1 hour. Metagene analysis of Pol II ChIP-Rx showed that THZ531 reduced Pol II occupancy at TSS, TES, and gene bodies in a time-dependent manner. **(E)** Meta-analyses of scaled LEO1-occupied genes with ratios of THZ531-treated Pol II to untreated Pol II. THZ531 treatment time-dependently decreased Pol II across the gene body, especially at the 3'-end of gene and TES regions. **(F)** Workflow for IP-MS of Flag-LEO1, Flag-LEO1 Δ540-666, and Flag-LEO1 Δ151-301. Flag-tagged LEO1 proteins were expressed in HEK293T cells and purified with M2 beads. The interacting proteins were precipitated with acetone and digested with trypsin. The resulting peptides were analyzed by LC-FAIMS-MS/MS and quantified by the Thermo Proteome Discoverer software. **(G)** Volcano plots of the abundance of LEO1 Δ151-301 vs the full-length LEO1 interacting proteins. **(H)** Immunoblotting of LEO1 in LEO1-HaloTag knockin cells expressing

Flag-LEO1 or LEO1 mutants with or without HaloPROTAC3 treatment. Tubulin serves as a loading control.

Fig S5. LEO1 C-terminus is required for processive transcription elongation.

(A) Pol II ChIP-Rx and PRO-seq analyses of the full-length LEO1 and LEO1 Δ540-666. Flag-tagged full-length LEO1 (WT) or LEO1 Δ540-666 were ectopically expressed in LEO1-HaloTag knockin cells, and HaloPROTAC3 was used to rapidly degrade the endogenous LEO1-HaloTag proteins before Pol II ChIP-Rx and PRO-seq analyses. **(B)** Heatmap analysis of ChIP-Rx with LEO1 and LEO1 Δ540-666 shows the deletion of LEO1 C-terminus (540-666) decreases genome-wide Pol II levels at the transcription units. **(C)** PRO-seq analysis of LEO1 and LEO1 Δ540-666 shows LEO1 C-terminal truncation globally reduces Pol II elongation and induces termination defects at the 3'-end of genes. Genes are sorted by length and shown from the TSS to 50 Kb. **(D)** Metagene plots showing LEO1 depletion decreases Pol II levels at promoters, gene bodies, and 3'-end of genes. **(E)** Metagene analysis of PRO-seq signals (r.p.m) in LEO1 WT and 3A cells. 3A mutant decreases the elongating Pol II at the gene body and TES regions. **(F** and **G)** Differential salt fractionation of nuclei to analyze chromatin-associated LEO1 proteins. LEO1-HaloTag knockin cells ectopically expressing LEO1 3A or WT were digested with MNase and extracted with different concentrations of salt. The extracted fractions were analyzed by immunoblotting with an anti-LEO1 antibody. **(H)** Alignment of LEO1 C-terminus sequences from 7 different species showing the level of conservation. Gaps are denoted as "-".

Table S1. Mass spectrometry analysis of in situ CDK12 phosphorylation substrates.

Table S2. Comparison of *in situ* CDK12 phosphorylation substrates with CDK2 and CDK9 substrates.

Table S3. Quantification of phosphorylation sites by THZ531 or 1-NA-PP1 treatments.

Table S4. List of additional oligonucleotides.
